# A large-scale electrophoresis- and chromatography-based determination of gene expression profiles in bovine brain capillary endothelial cells after the re-induction of blood-brain barrier properties

**DOI:** 10.1186/1477-5956-8-57

**Published:** 2010-11-15

**Authors:** Gwënaël Pottiez, Barbara Deracinois, Sophie Duban-Deweer, Roméo Cecchelli, Laurence Fenart, Yannis Karamanos, Christophe Flahaut

**Affiliations:** 1 Univ Lille Nord de France, F-59000 Lille, France; 2 UArtois, LBHE, F-62307 Lens, France; 3 IMPRT-IFR114, F-59000, Lille, France

## Abstract

**Background:**

Brain capillary endothelial cells (BCECs) form the physiological basis of the blood-brain barrier (BBB). The barrier function is (at least in part) due to well-known proteins such as transporters, tight junctions and metabolic barrier proteins (e.g. monoamine oxidase, gamma glutamyltranspeptidase and P-glycoprotein). Our previous 2-dimensional gel proteome analysis had identified a large number of proteins and revealed the major role of dynamic cytoskeletal remodelling in the differentiation of bovine BCECs. The aim of the present study was to elaborate a reference proteome of Triton X-100-soluble species from bovine BCECs cultured in the well-established *in vitro *BBB model developed in our laboratory.

**Results:**

A total of 215 protein spots (corresponding to 130 distinct proteins) were identified by 2-dimensional gel electrophoresis, whereas over 350 proteins were identified by a shotgun approach. We classified around 430 distinct proteins expressed by bovine BCECs. Our large-scale gene expression analysis enabled the correction of mistakes referenced into protein databases (e.g. bovine vinculin) and constitutes valuable evidence for predictions based on genome annotation.

**Conclusions:**

Elaboration of a reference proteome constitutes the first step in creating a gene expression database dedicated to capillary endothelial cells displaying BBB characteristics. It improves of our knowledge of the BBB and the key proteins in cell structures, cytoskeleton organization, metabolism, detoxification and drug resistance. Moreover, our results emphasize the need for both appropriate experimental design and correct interpretation of proteome datasets.

## Background

The endothelia of different organs are remarkably heterogeneous but do present many common functional and morphological features. Given the endothelium's strategic position between the blood and the tissues, this cell layer (i) closely controls the transport of plasma molecules (via bidirectional receptor-mediated and receptor-independent transcytosis and endocytosis), (ii) regulates vascular tone, (iii) synthesises and secretes a wide variety of factors and (iv) is involved in the regulation of inflammation, haemostasis, thrombosis and immunity. It is now also generally accepted that the specific ultrastructure of capillaries in the brain, retina, kidney and liver governs the specialized physiological properties of these respective endothelia [1]. In the brain, the blood-brain barrier (BBB) separates the brain microvasculature from the peripheral microvasculature. The BBB constitutes a physical and metabolic barrier which tightly regulates blood-brain exchanges of ions, small molecules and proteins and is involved in the recruitment of immune cells prior to transfer to the brain during inflammation [2-4].

In brain capillaries, the BBB is formed by endothelial cells, which are surrounded by a tubular sheath of astrocytic end-feet. Pericytes are inserted into the basal membrane (between the endothelium and the astrocytic end-feet) [3]. This spatial cell layout and the resulting astrocyte-endothelium communication induce the establishment and maintenance of the BBB [5-7]. Dysregulation of these processes has been linked to the pathogenesis of several human diseases [8].

In the brain, only blood capillaries are endowed with a complete BBB phenotype [9]. Under physiological conditions, the barrier function is performed by a number of unique endothelial features, including (i) the lack of fenestration, (ii) a decrease in the number of pinocytic vesicles, (iii) the reinforcement of complex tight junctions and (iv) the upregulated expression of metabolic enzymes and plasma membrane transporters and receptors [5]. The physiological consequences of endothelial cell differentiation include an increase in the transendothelial electrical resistance (due to a decrease in the para- and transcellular endothelial permeability of ions and low-molecular-weight hydrophilic compounds) and are associated with marked polarization of the cerebral endothelium [10,11]. In brain endothelial cells, the plasma membrane acts as the controlling interface for intracellular molecular signalling, the reinforcement of tight junctions and molecular and cell transport between the brain and the blood. The plasma membrane of brain capillary endothelial cells (BCECs) has been extensively studied and its membrane protein expression pattern has been well defined [12]. The intracellular location of certain proteins was shown to be essential for the establishment and maintenance of the BBB-related features of BCECs. These intracellular locations are frequently used as quality control criteria for *in vitro *BBB models. Furthermore, it is known that the protein distribution changes under pathological conditions [13,14]. Paradoxically, no dedicated studies in this field have been reported. Moreover, the BBB's metabolic proteome is not well known and the cytosolic, nuclear and mitochondrial protein expression profiles have yet to be extensively characterized. Therefore, the use of Triton X-100 (known to poorly solubilise sparingly soluble proteins [15]) appeared as the best way to select the BCECs' cytosolic subproteome in the present study.

Proteomics deals with the direct, large-scale determination of gene and cellular function at the protein level. Recent successes have emphasized the role of mass spectrometry-based proteomics as an essential tool in molecular and cellular biology. Two-dimensional polyacrylamide gel electrophoresis (2-DE) has become the core technology for building proteomic databases, for several reasons. Firstly, 2-DE can display thousands of proteins and allows the relative quantification of any given polypeptide. Secondly, the high-mass shifts caused by one or more posttranslational modifications can be characterized. Thirdly, digitized 2-DE images are convenient, informative visual media for creating a web-based database. Although 2-DE can (in theory) separate all proteins, the technique has a number of drawbacks in practice: (i) it is difficult to separate proteins with low (< 10 kDa) or high (> 200 kDa) molecular weights, (ii) the truly operational *p*I range is 3-11 and (iii) protein spots are frequently superposed. Complementary approaches are often needed to overcome incompatibilities between the 2-DE separation technique and the physical-chemical properties of certain proteins of interest. Liquid chromatography (LC) is an alternative (or a complementary counterpart) to 2-DE.

On the basis of the *in vitro *BBB co-culture model developed in our laboratory [16], we have initiated a comprehensive proteomic approach based on the extractability of proteins with Triton X-100. It uses a 2-DE/1D-LC combination to provide a detailed expression profile for the Triton X-100-soluble proteins expressed by bovine BCECs (BBCECs) with the BBB phenotype. Here, we report on the use of a high-throughput proteomics platform in the 2-DE identification of the most abundant Triton X-100-soluble proteins, together with additional 1D-LC identification. In addition to investigation of the correlation between the identified proteins and BBB functions, the present study was also designed to prime a comprehensive, interactive, web-based proteomics database for BCECs (2-DE images, comparative results, mass spectra, identification scores, protein identities, protein functions, etc.), in accordance with published guidelines [17,18].

## Results

### Verification of the re-induction of BBB properties

When isolated *in vitro*, primary BBCECs dedifferentiate and lose their BBB properties. The latter can be restored by 12 days of co-culture with glial cells [16]. We confirmed the re-induction of BBB properties by (i) measuring an optimal value for the paracellular permeability coefficient (Pe) and (ii) immunostaining the main tight junction proteins (ZO-1, occludin and claudin-5). As reported in the literature [19,20], the markers display a pericellular distribution in differentiated BBCECs, which contrasts with the uniform pattern for dedifferentiated cells (data not shown). The BBCECs were harvested and lysed according to the procedure described by Pottiez *et al*. [21]. The Triton X-100-solubilised protein fraction (representing 85% of the cell's total protein) was then subjected to either 2-DE or off-line 1D-LC tandem mass spectrometry (MS/MS) analysis with a C18 column.

### Two-dimensional polyacrylamide gel electrophoresis

**Figure 1:**
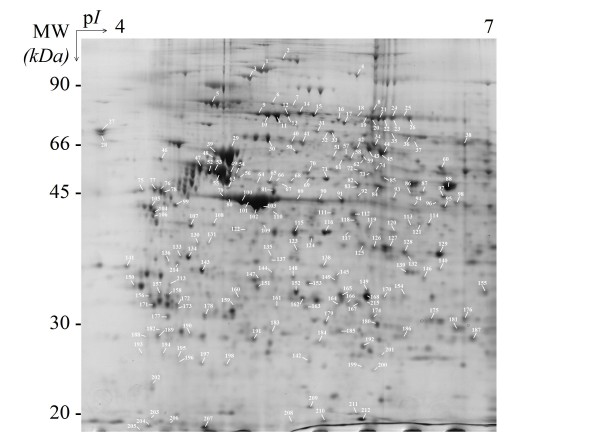
**Gel image from the 2-DE of Triton X-100-extracted proteins from bovine brain capillary endothelial cells displaying the blood-brain barrier properties**. 215 protein identities are reported on this 2-DE gel image and are summarized in Table S1 (see Additional File 1).

Proteins of interest were subjected to 2-DE over a pH range of 4-7 and a molecular weight range of 100-10 kDa. Four gels were prepared from four different filter inserts. The 2-DE-separated proteins were stained with either silver nitrate (to evaluate the reproducibility of 2-DE separation) or Coomassie brilliant blue (to perform the subsequent protein identification steps). Next, 384 of the most intense, best-resolved polypeptide spots were automatically identified according to a Proteineer™workflow. Of the 384 excised spots, 215 (56%), were unambiguously identified (Figure 1, in which the numbering corresponds to identified proteins; non-identified proteins are not reported) and 198 were identified as bovine proteins. Protein identifications were mainly based on the peptide mass fingerprint (PMF) principle, in which the Mascot 2.2 identification algorithm was used to search the NCBInr and Swiss-Prot 57.7 databases. The Mascot scores from the two databases were similar for all but 9 proteins (indicated by an asterisk in the Table S1, see Additional File 1). Moreover, 85 protein identities were confirmed by peptide fragmentation fingerprinting (PFF). The 215 identified spots corresponded to 130 distinct proteins (grouped together in Table S1 in alphabetical order for convenience and clarity). Interestingly, 8 protein spots (#42, 61, 90, 91, 113, 121, 127 and 147) were identified as containing two proteins. Proteins with similar primary amino acid sequences (e.g. ezrin-radixin-moesin complex proteins) were well resolved by 2-DE and, despite their sequence homologies, were more conveniently identified by PMF than by PFF (data not shown).

### One-dimensional liquid chromatography

**Figure 2:**
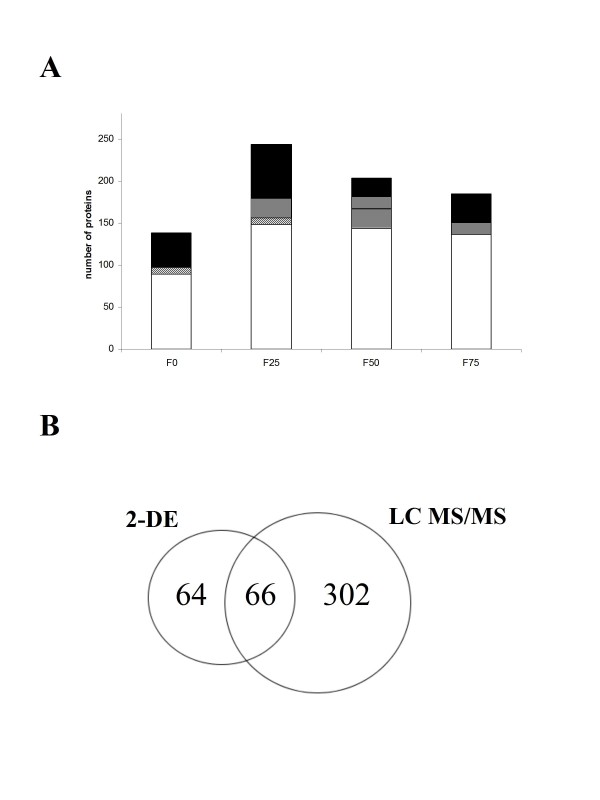
**Cross-analysis of protein lists**. The sorting and cross-analysis of protein lists was performed using nwCompare software. (A) a comparative histogram of 1D-LC analyses of the main fractions. Black areas: fraction-specific proteins; white areas: proteins found in at least three different fractions; diagonal hatching: the proportion of proteins common to the F0 and F25 fractions; vertically hatching: the proportion of proteins common to F25 and F50 fractions; horizontally hatching: the proportion of proteins common to the F50 and F75 fractions. (B) a Venn diagram showing the complementarity of the two approaches (in terms of the proteins identified).

Twenty μg of Triton X-100-solubilized proteins from BBCECs were subjected to a 16-hour in-solution digestion. The mixture of trypsin-generated peptides was resolved by 1D-LC (on a C18 column with a linear acetonitrile gradient), spotted and concomitantly co-crystallised with α-cyano-4-hydroxycinnamic acid (CHCA) matrix on a matrix-assisted laser desorption/ionization (MALDI) target. The 380 spotted fractions underwent automatic MS and MS/MS measurements. Around 12000 compounds were detected in the MS step and 3000 of these were subjected to MS/MS fragmentation. Due to the insufficient separative power of the 1D-LC protocol, only a few peptides yielded a fragmentation pattern which was suitable for identification (data not shown). Since 2D-LC technology is unavailable in our laboratory, we developed a fractionation step (see Material and Methods) for reducing protein heterogeneity prior to a 1D-LC analysis and for ensuring as many protein identifications as possible. Each subfraction was subjected to the previously described off-line 1D-LC separation, in which around 3000 components were MS-detected in the main fractions (F0, F25, F50 and F75). Around 400 of these components were in-source fragmented and 250 MS/MS spectra provided unambiguous protein identities. An average of 138 proteins were identified from the F0 subfraction, with 235 in the F25 subfraction, 180 in the F50 subfraction, 185 in the F75 subfraction and only 9 from the F100 subfraction (Figure 2A). The latter figure also shows the proportion of proteins specifically identified in only one fraction; fractions F0, F25, F50 and F75 contained 41, 64, 22 and 35 fraction-specific proteins, respectively (black areas). Moreover, 8 proteins were found in fractions F0 and F25 (Figure 2A, diagonally hatched area), 23 proteins were common to fractions F25 and F50 (Figure 2A, vertically hatched area) and only 14 proteins were identified in both fractions F50 and F75 (Figure 2A, horizontally hatched area). Ultimately, no fewer than 363 proteins (all listed in Table S2, see Additional File 2) were identified; 1D-LC MS/MS data are available in the European Bioinformatics Institute's PRIDE database [22]http://www.ebi.ac.uk/pride under accession numbers 12825 to 12830. The data were converted using the PRIDE Converter [23]. In all, 134 proteins (around 37%) were identified on the basis of only one peptide sequence, with 103 proteins identified from 2 MS-fragmented peptides and 183 proteins identified from between 3 and 10 fragmented peptides. Nineteen proteins (such as vimentin, titin, meosin, alpha-actinins 1 and 3, filamins A and B, clathrin heavy chain 1, ATP synthase beta-subunit, vinculin, spectrin alpha chain (brain isoform) and many tubulins (beta, beta-2C, beta-4, beta-5, etc.)) were identified with 10 to 25 peptides sequenced. These multi-sequenced proteins were mostly either abundant cell proteins or high-mass proteins. As shown in Figure 2B, a comparison of the protein sets identified respectively by 2-DE or 1D-LC emphasizes the complementarity of the two techniques; only 66 proteins (15%) were identified by both techniques, whereas the great majority of the proteins (302) were identified by 1D-LC.

### Molecular functions of the identified proteins and their involvement in biological processes

**Figure 3:**
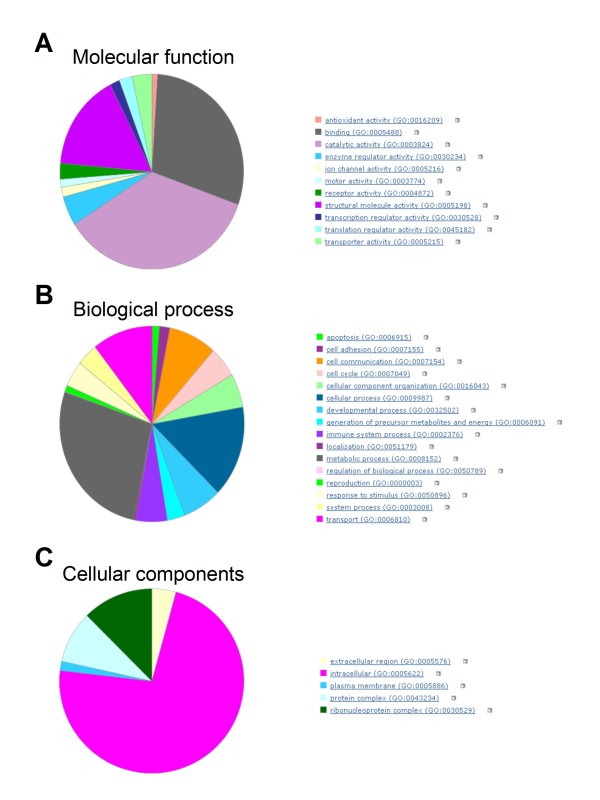
**Chart of biological processes and molecular functions inherent to the identified proteins**. Classification of all identified proteins was performed using the *Protein Analysis Through Evolutionary Relationships *(PANTHER) classification system http://www.pantherdb.org. Proteins are classified by expert biologists into families and subfamilies of shared function, which are then categorized by molecular function (A) and biological process (B) ontology terms or in regard to the cellular components (C).

In order to classify all identified proteins according to their molecular functions and the biological processes in which they are involved, the correspond gene names were generated using DAVID bioinformatics resources http://david.abcc.ncifcrf.gov/home.jsp. All but 6 of the 430 genes were successfully classified according to the PANTHER system http://www.pantherdb.org; ERP44, ESYT1, Hist1h4a, NOP56, PLDB2 and TBA1B failed, for unexplained reasons. The identified proteins displayed 11 molecular functions and are involved in 16 biological processes (Figures 3A and 3B). As expected, most of the identified species were metabolic proteins, binding proteins or cell structure proteins (i.e. cytoskeletal proteins or those involved in cytoskeleton formation and regulation). Interestingly, we also identified proteins associated with the transport and cell-cell communication (Figures 3A and 3B). Lastly (and as expected), 95% of the identified species were soluble (i.e. only 5% were plasma membrane proteins) and 75% were intracellular proteins (Figure 3C.).

### The protein interference problem in a gel-free approach

**Figure 4:**
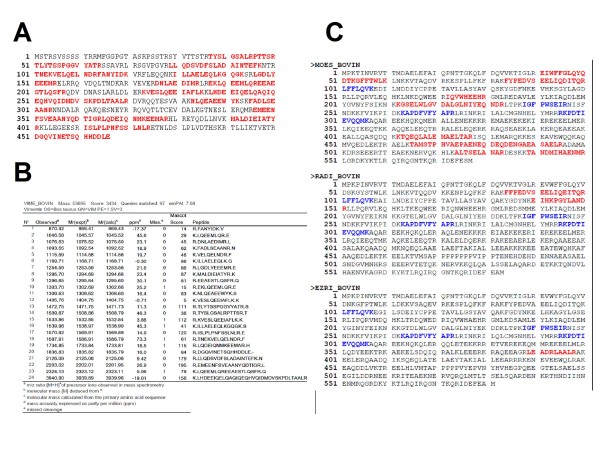
**Protein-level evidence of the expression of vinculin within bovine brain capillary endothelial cells**. (A) A screen print from the Mascot results page, showing the sequence coverage obtained for bovine vimentin. (B) A detailed overview of the MS/MS sequenced peptides from bovine vimentin. (C) Sequence coverage for proteins displaying similar primary amino acid sequences: MOES_BOVIN, Moesin; RADI_BOVIN, radixin; EZRI_BOVIN, ezrin (components of the ezrin-radixin-moesin (ERM) complex). The peptides common to all sequences are highlighted in blue and the amino acid sequences which are specific for a given protein are shown in red.

In all, 2-DE identified 29 protein spots for vimentin (from 55 to 40 kDa and with *pI *values of between 5 and 4), whereas 1D-LC identified vimentin as a unique protein with 24 mass-sequenced peptides and a sequence coverage of 56% (Figures 4A and 4B). In a shotgun approach, proteins with similar primary amino acid sequences (e.g. ezrin-radixin-moesin complex proteins) were identified on the basis of their specific peptides. The more abundant moesin was identified with 8 specific peptides, whereas radixin and ezrin were identified with only two and one specific peptides, respectively (Figure 4C).

### Evidence for the existence of a 1134-amino acid bovine vinculin

**Figure 5:**
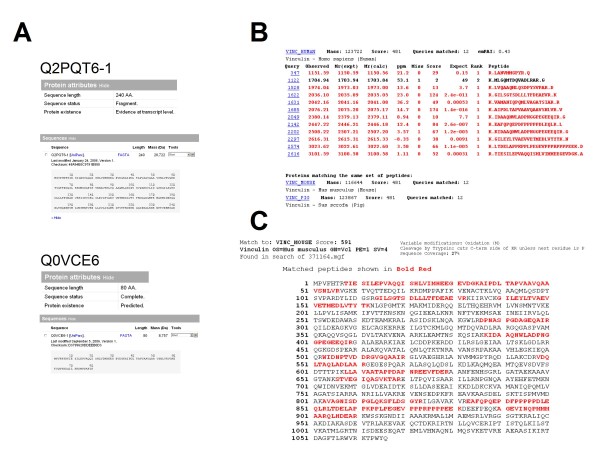
**Additional evidence for the expression of vinculin within bovine brain capillary endothelial cells**. (A) A screen shot from UniprotKB for bovine vinculin files. (B) A screen shot of the MASCOT results page following a search against the 57.7 UniprotKB database, showing the 12 sequenced peptides matching the human vinculin amino acid sequence. (C); A screen shot from the MASCOT results page showing the distribution of peptides over the full-length amino acid sequence of the predicted bovine vinculin (NW_001501727).

In the present study, we clearly identify vinculin with 1D-LC approach. However, the bovine forms (Q2PQT6-1 and Q0VCE6) listed in UniprotKB (Figure 5A) were not reported in the MASCOT result page (Figure 5B). Fortunately (thanks to inter-species sequence homology), the identifying result (Figure 5B) indicated that the vinculin was from human, mouse and pig (with an identical cumulative Mowse score of 481 for all species). As shown in Figure 5B, 12 mass-sequenced peptides matched the amino acid sequence of human vinculin (only 3 MS/MS-sequenced peptides display a low MASCOT score, due to low-quality fragmentation spectra). In contrast, the MASCOT submission of PFF dataset to the NCBInr database gave a positive match (Figure 5C) for a predicted bovine vinculin (XP_001790344). The 12 mass-sequenced peptides were homogeneously distributed from the N-terminal to the C-terminal. This record is derived from a genomic sequence (NW_001501727) which was annotated using the GNOMON gene prediction method and is supported by EST evidence. This example serves as a reminder that to ensure high-quality protein identification, the simultaneous submission of a proteomics dataset to at least two protein databases is as important as the use of a decoy database.

## Discussion

The battle against the relentless progression of neurodegenerative disease, brain tumours and brain damage is a public health priority. Although effective drugs have been reported on the basis of *in vivo *research results, drug targeting and delivery to the brain are still constitute a challenge [24] one century after the discovery of the BBB. In fact, the BBB is often the rate-limiting factor in drug transport into the brain. Models of the BBB are of major importance in efforts to expand our understanding of how the brain maintains its integrity and controls the inward and outward fluxes of endogenous substances and drug compounds [9]. The development of genomic and proteomic technologies has provided several means of extending our knowledge of the BBB and investigating additional routes for bypassing this barrier [4].

Several BCEC-dedicated whole-cell or plasma membrane proteomic approaches have been reported. Most have been comparative studies designed to detect and identify candidate proteins which are either differentially expressed in two distinct conditions [25,26], involved in a given physiological process [21,27] or expressed in response to a particular stimulus [14,28-31]. Very few studies have focused on sub-proteomes such as the protein content of the plasma membrane [12] or that of the caveolae [32]. Although this type of targeted proteomic study is essential for unravelling the molecular mechanisms governing the physiology and physiopathology of the BBB's endothelial cells, only small sets of protein identities tend to be reported. Hence, large-scale determinations of expression profiles for a given protein class or cell compartment-related protein subset in BBB-differentiated BCECs (relative to non-brain capillary endothelial cells) have yet to be performed in any species. The large-scale determination of BCEC protein contents is a essential analytical step in gaining a better understanding of the physiopathology of the BBB [33].

In the field of BBB research, proteomic analyses of BCECs displaying BBB features and functions often focus on proteins related to the tight junction, the adherens junction, transport, drug resistance and the regulation of the latter functions [34]. Most of the aforementioned proteins are transmembrane proteins anchored to the actin cytoskeleton by cytosolic proteins. The importance of the intracellular location has been well demonstrated [13]. In general, cytosolic proteins are ignored - despite the fact that they are known to play a role in many BBB functions. Our present study provided a partial list of non-characterized cytosolic proteins with possible relevance in BBB function. Firstly, many of the identified proteins (27%) are related to metabolic processes, such as the primary metabolism of proteins, carbohydrates, nucleobases/nucleosides/nucleotides, lipids, amino acids and their derivatives. We consider that knowledge of the BCECs' enzymatic equipment constitutes a breakthrough. By way of an example, levels of nitric oxide (known to modulate BBB permeability) are related to the metabolism of arginine [35]. Oxidative stress is also an important modulator of BBB permeability. In the present study, we showed that 31 oxidoreductase proteins are expressed by BCECs. Secondly, binding proteins represent around 30% of the identified set. Of these, 32 are known to interact with Ca^2+^. It has been reported that both increases and depletion of intracellular Ca^2+ ^may result in disruption of intercellular junctions [36]. We also detected 104 nucleotide-binding proteins including 34 ribonucleoproteins and 38 RNA-binding proteins (ribosomal proteins, heterogeneous nuclear ribonucleoproteins, splicing factors, eukaryotic translation initiation factors, tRNA synthetase, etc.). Furthermore, 73 and 30 of the identified proteins are respectively ATP- and GTP-binding proteins. Sixty-five proteins are described as having molecular transport activities. Thirdly, structural components account for around 20% of the proteins identified: half of the latter (n = 44) are cytoskeleton components or cytoskeleton-associated proteins. In addition to the presence of proteins like ERM complexes, filamins, heat shock proteins, plectin, plastin 3, alpha-actinins, cofilins, actin-capping proteins, spectrins and tropomyosins, we report the expression of (i) junction plakoglobin (gamma catenin), a cytoplasmic protein in soluble and membrane-associated forms and (ii) Talin 1, a crucial protein for reinforcement of integrin-cytoskeleton bond and which binds vinculin with high affinity [37]. Fourthly, 45 and 37 proteins reported in this study are from mitochondria and endoplasmic reticulum and which are involved not only in metabolism but also in cell function. One example is the tumour necrosis factor receptor-associated protein 1 (TRAP-1) which protects cells from oxidative stress and apoptosis [38].

*In fine*, 293 of the identified proteins are phosphoproteins. Hence, Triton X-100-dependent protein extraction may constitute a way of enriching the phosphoproteome subset.

The creation of a dedicated proteomic database is the best way of gathering and storing proteome information but does require perfect sample traceability and strict compliance with protein identification guidelines [17] in order to avoid human error and, with a view to high throughputs, guarantee the identity of proteins by reducing the false positive rate as much as possible. Likewise, the repeatability of protein identifications (at least triple mass measurement of the same spot) must be carefully checked and not postponed within the database to assure the latter's clarity and avoid obstruction (especially in the case of publicly available databases). Due to the inherent drawbacks of each analytical technique, the use of several different approaches is the best way of ensuring the broadest possible determination of proteins from a given proteome. The recent development of commercially available integrated solutions for high-throughput protein identification (combining bio-informatics tools and automated sample handling with one or several mass spectrometers) constitutes a true source of progress; it saves time, reduces the number of errors, increases the analytical throughput, centralizes the proteomic data and enables more efficient comparison of datasets. Nevertheless, most in-house 2-DE, 1D-LC or 2D-LC databases are not publicly available (for various reasons). As stated in a recent review article [39,40], this situation can easily be resolved by public, community-based solutions for the long-term storage, management, sharing and comparison of proteomic data, such as MIAPEGelDB [41], the World-2DPAGE repository [42], PRIDE [43,44], GPMDB [45], Peptideatlas [46] and PrestOMIC [47]. However, we believe that a number of limitations remain.

The expansion of the LC-MS proteomic data storage is significantly greater than that of the 2-DE proteomic data. We believe that this can be explained by several factors. Firstly, shotgun strategies are remarkably automated and powerful and clearly increase the overall data throughput compared with 2-DE gel. Secondly, the web-submission process in the proteomic repositories of LC-MS proteomic data is now automatized, convenient and secure (mainly thanks to the use of converters, such as the PRIDE converter). Thirdly, web submission of 2D-PAGE proteomic data is still a manual, fastidious, time-consuming step and is prone to human error. It therefore requires more preparation steps (for the 2-DE gel pictures, spot location data, MS data, MS protein identification data, etc.); this situation clearly weakens the commitment to communal sharing and prompts researchers to overuse shotgun approaches.

The diversity of MS file formats is now handled well by almost all proteomic data repositories, although some proprietary file formats developed by MS manufacturers are not yet supported. Therefore, proteomic data can be submitted over the web via protein identification result files, such as Mascot .dat files and SEQUEST result files. However, whereas the MS/MS files used to create the PFF are fully supported, the files corresponding to protein identifications based on PMF or combined PMF/PFF protocols are not supported and the automatic web-submission of the latter two files types is still not possible. Overall, these are probably the main limiting factors on the expansion of public submission of 2-DE proteomic datasets. We have contacted Dr Juan Antonio Vizcaíno in the PRIDE group with a view to achieving the parsing of PMF and combined PMF/PFF Mascot data files from 2-DE experiments.

Protein databases are now so large that syntactic errors are now acknowledged to be one of the reasons for absent or false protein identifications. For example, bovine vinculin is reported in UniprotKB under two accession numbers Q2PQT6 and Q0VCE6 with 240 and 80 amino acids respectively. However, unmatured vinculin displays an average of 1134 amino acids for isoform-1 and 1066 for isoform-2 from distinct species (human, mouse, rat and pig). The NCBInr database reports a bovine vinculin sequence (NW_001501727) derived from a gene prediction process. Hence, on the basis of NCBInr database and our LC-MS/MS data, we identified a 1134- amino acid bovine vinculin; this shows that vinculin is expressed in BBCECs in a form similar to those reported in mammals. This type of *de novo *sequencing information (i) provides direct evidence of the existence of listed proteins in a given cell type in a given species, (ii) enables the correction of certain mistakes reported in protein databases (e.g. bovine vinculin) and (iii) can confirm predictions generated by genome annotation methods.

Vimentin identification is a classic example of the technological bias inherent in each proteomic strategy. Whereas 2-DE is able to distinguish between several vimentin isoforms (resulting from post-translational modifications), 1D-LC identifies only one protein. Vimentin is one of the most prominent phosphoproteins in various mesenchymal cells in which phosphorylation is enhanced during cell division [48], when vimentin filaments are significantly reorganized. Phosphorylation also determines the assembly dynamics of vimentin intermediate filaments [49]. Vimentin is also subject to acetylation. In all, more than 50 isoforms are listed in the protein databases. Consequently, vimentin cannot be simply considered as the single protein reported by 1D-LC. The 1D-LC identification of proteins with very similar primary amino-acid sequences is far from perfect because it is based on the PFF corresponding to protein-specific peptides. This is known as the protein interference problem [50] and, unfortunately, is still ignored in too many cases. For soluble proteins > 20 kDa, the PMF obtained from 2-DE is more likely to unambiguously distinguish between paralogous proteins. Ultimately, combined PFF/PMF datasets are more discriminant and have a greater certainty of identification because they are derived from in-gel separated proteins.

## Conclusions

Our combination of 2-DE and 1D-LC approaches enabled the first ever identification of about 430 Triton X-100-soluble proteins (see Additional File 3) from BBCECs displaying BBB characteristics. The information on these protein identifications is now stored in in-house databases and will be soon shared through the PRIDE database for convenient comparison with proteomic datasets from non-brain vascular endothelial cells. Due to the extraction conditions, fewer than 5% of the proteins corresponded to membrane-associated proteins. More than 75% of the identified proteins display binding, catalytic or structural functions. Most identified proteins were involved in metabolic and cellular processes but transport and cell-cell communication process accounted for almost 25% of the identified species. The present study also emphasized the advantage of inter-species sequence homology comparisons for protein identification in non-completely sequenced genomes and highlighted the drifts and drawbacks generated by the rapid, gel-free proteomic methods (which nevertheless provide most of today's data). In the absence of a physiological, human *in vitro *BBB model, the proteomics expression profile of BBCECs displaying BBB properties is the first step towards the creation of an anti-BBB antibody library which will greatly facilitate large-scale, array-based screening of protein expression.

## Methods

### Materials

Heat-inactivated calf serum and horse serum were bought from Hyclone Laboratories (Logan, UT, USA). Glutamine and gentamycin were purchased from Biochrom AG (Berlin, Germany). Serum, basic fibroblast growth factor and Dulbecco's Modified Eagle's Medium (DMEM) were from GIBCO (Invitrogen Corporation, Carlsbad, CA, USA). Six-well plates and Transwell inserts were from Corning Inc. (New York, NY, USA). The duracryl/bis-acrylamide solution was from Genomic Solution (Proteomic Solutions, Saint-Marcel, France). Immobilized pH gradient-ready strips were from GE Healthcare (Amersham Bioscience, Orsay, France). Trypsin digestion kits for the DP Proteineer™robot and alpha-cyano-4-hydroxycinnamic acid were from Bruker Daltonik GmbH (Bremen, Germany). All other reagents were of analytical or electrophoresis grades.

### Cell culture and the blood-brain barrier model

Primary cultures of mixed glial cells were made from newborn rat cerebral cortex, as described by Booher and Sensenbrenner [51]. Briefly, glial cells were cultured in DMEM supplemented with 10% (v/v) heat-inactivated foetal calf serum, 2 mM glutamine and 50 μg/ml gentamycin. Three weeks after seeding, the glial cell cultures were stabilized and used for co-culture. Bovine brain capillary endothelial cells were isolated and characterized as described by Méresse *et al*. [52]. The BBCECs were co-cultured until confluence (12 days) with glial cells on an extracellular matrix (rat tail collagen) in DMEM supplemented with 10% (v/v) heat-inactivated calf serum, 10% (v/v) horse serum, 2 mM glutamine, 50 μg/ml gentamycin and 1 ng/ml basic fibroblast growth factor.

### Cell harvesting and protein extraction

The endothelial cells (8 × 10^5 ^cells) were harvested by collagenase treatment (*Clostridium histolyticum*, Sigma, Lyon, France) [21]. Briefly, the cells were treated at 37°C for 40 min with 1.5 ml of a collagenase solution (0.1% w/v). The collected cell materials were rinsed 3 times in phosphate buffered saline (PBS), centrifuged 10 min at 500 g. The cell pellets were lysed in 200 μl of lysis buffer [Tris/HCl 10 mM, EDTA 1 mM, Triton X-100 1% (v/v), 2-mercaptoethanol 0.1% (v/v) and protease inhibitors (Roche Biomoleculars, Meylan, France)] and centrifuged (13,500 g, 4°C, 45 min). The protein content of the Triton X-100 soluble and insoluble fractions was assessed [53]. The supernatants were concentrated, desalted and delipidated by overnight acid precipitation at -20°C.

### Two-dimensional gel electrophoresis

After resolubilization in an isoelectrofocusing buffer (7 M urea, 2 M thiourea, 4% (v/v) CHAPS and 2% (v/v) ampholytes), 300 μg of cytosolic proteins were subjected to 2-DE on 24-cm length pH 4-7 IPG strips at 100,000 V and in the 10-100 kDa molecular mass range. The IPG strips were passively and actively rehydrated for 7 h and 9 h at 50 V. The pre-focusing and focusing procedures were carried out at 50 mA/strip in 4 steps: 200 V for 1 h, a gradient up to 1000 V for 1 h, a gradient up to 10000 V for 6 h and, lastly, 10000 V for 4.5 h. The IPG strips were wiped up and successively equilibrated for 15 min with gentle shaking in 6 M urea, 20% (v/v) glycerol, 2% (w/v) SDS, 93 mM TRIS-HCl pH 8.8 buffers supplemented with 20 mM DTT and 100 mM iodoacetamide and a trace of BPB, respectively. The equilibrated strips were sealed on the top of the second-dimension duracrylamide/bis-acrylamide gel (12% T, 2.6% C) with 0.5% (w/v) low-melting point agarose (Biorad, Marnes-la-Coquette, France) in SDS running buffer. Migration as a function of molecular weight was performed in the Ettan DALTsix electrophoresis unit (Amersham Bioscience) at 16 mA/gel for 30 min and then at 32 mA/gel until the tracking dye reached the anodic end. The proteins were stained with silver nitrate [54] for image acquisition (with a freshly calibrated Umax scanner (Amersham Biosciences, Orsay, France) at 300 dpi using Labscan 3.0 software) and with colloidal Coomassie Brilliant Blue for protein identification by MALDI-TOF/TOF mass spectrometry.

### Protein identification experiments after 2D-PAGE

Protein identifications from 2-DE gels were performed using a Proteineer™workflow from Bruker Daltonik GmbH. Colloidal Coomassie-blue-stained spots were excised from gels with a spot picker (the Proteineer™spII) equipped with a 2 mm needle and placed into 96-well microtitre plates. In-gel digestion and sample preparation for MALDI analysis were performed according to the manufacturer's instructions using a digester/spotter robot (the Proteineer™dp) and tryptic digest kits (the DP 384 standard kit from Bruker Daltonik). Briefly, after destaining of the gel plugs with 10 mM ammonium bicarbonate and 50% acetonitrile in 10 mM ammonium bicarbonate, protein spots were digested essentially according to Shevshenko *et al*., 1996 [54]. Peptide were extracted with acetonitrile: 0.1% TFA-acidified water (1:1) and then mixed with an α-cyano-4-hydroxycinnamic acid matrix (0.3 mg/ml in acetone:ethanol, 3:6 v/v) on the MALDI target plate (AnchorChip™, Bruker Daltonics). The molecular mass measurements were performed in automatic mode using FlexControl™ 2.2 software on an Ultraflex™ II TOF/TOF instrument and in reflectron mode for MALDI-TOF PMF or LIFT mode for MALDI-TOF/TOF PFF. External calibration over the 1000-3500 mass range was performed using the [M+H]^+ ^monoisotopic ions of bradykinin 1-7, angiotensin I, angiotensin II, substance P, bombesin and adrenocorticotropic hormone (clips 1-17 and clips 18-39) from a peptide calibration standard kit (Bruker Daltonik). Briefly, a 25 kV accelerating voltage, a 26.3 kV reflector voltage and a 160 ns pulsed ion extraction were used to obtain the MS spectrum. Each spectrum was produced by accumulating data from 500 laser shots. Two precursor ions per sample at most were chosen for LIFT-TOF/TOF MS/MS analysis. Precursor ions were accelerated to 8 kV and selected in a timed ion gate. Metastable ions generated by laser-induced decomposition (LID) were further accelerated by 19 kV in the LIFT cell and their masses were measured in reflectron mode. Peak lists were generated from MS and MS/MS spectra using Flexanalysis™ 2.4 software (Bruker Daltonik). Database searches with Mascot 2.2 (Matrix Science Ltd, London, UK) using combined PMF and PFF datasets were performed in the UniProt 57.7 and NCBInr databases via ProteinScape 1.3 (Bruker Daltonik). A mass tolerance of 75 ppm and 1 missing cleavage site for PMF and an MS/MS tolerance of 0.5 Da and 1 missing cleavage site for MS/MS searching were allowed. Variable cysteine carbamidomethylation and methionine oxidation were also considered. The relevance of protein identities was judged according to the probability-based Mowse score, calculated with *p *< 0.05.

### NanoLC-MALDI-TOF-MS/MS experiments

Triton-soluble proteins from differentiated BBCECs are extremely heterogeneous. To attenuate this phenomenon, we fractionated the samples into 5 fractions of increasing concentration in acetonitrile (0%, 25%, 50%, 75% and 100%). Following the tryptic digestion, nanoseparations were performed on an U3000 nanoHPLC system (Dionex-LC-Packings, Sunnyvale, CA, USA). After a conventional pre-concentration step (C18 cartridge, 300 μm, 1 mm), the peptide samples were separated on a Pepmap C18 column (75 μm, 15 cm) using an acetonitrile gradient from 5% to 12% over 20 minutes, 12% to 50% over 140 minutes and 50% to 70% over 15 minutes and, lastly, 15 minutes in 70% of acetonitrile. The flow was set to 300 nl/min and 380 fractions were automatically collected every 30 seconds on an AnchorChip™ MALDI target using a Proteineer™FC fraction collector (Bruker Daltonik). 2 μl of CHCA matrix (0.3 mg/ml in acetone:ethanol:0.1% TFA-acidified water, 3:6:1 v/v/v) were added during the collection process. The MS and MS/MS mass measurements were performed off-line using the Ultraflex™ II TOF/TOF mass spectrometer, as described above. The apparatus parameters were set to the values given above. Peptide fragmentation was driven by Warp LC software (Bruker Daltonik) according to the following parameters: signal-to-noise ratio > 15, more than 3 MS/MS by fraction if the MS signal was available, 0.15 Da of MS tolerance for peak merge and the elimination of peaks which appears in more than 35% of the fractions. The protein identification was performed as described above.

### Sorting protein lists and bioinformatics resources

The protein lists were compared using nwCompare software [55]. All identified proteins were converted into gene names with the DAVID bioinformatics resources [56] before to be classification by the *Protein Analysis Through Evolutionary Relationships *(PANTHER) classification system. PANTHER is a unique resource that classifies genes by their functions, using published scientific experimental evidence and evolutionary relationships. Proteins are classified by expert biologists into families and subfamilies of shared function, which are then categorized by molecular function and biological process ontology terms [57,58].

## Competing interests

The authors declare that they have no competing interests.

## Authors' contributions

CF designed the study and participated in its initiation and coordination, together with SDD. Cell culture was managed by GP and BD. GP and BD carried out the 2DE-map and nanoHPLC experiments and helped draft the manuscript. YK, RC and LF participated in the study design and helped draft the manuscript. All authors have read and approved the final manuscript.

## Supplementary Material

Additional File 1 **Table S1**. File reporting the complete gene list identified from the 2D-PAGE experiment. Click here for file

Additional File 2 **Table S2**. File corresponding to the complete gene list identified from the LC-MS experiment. Click here for file

Additional File 3 **The complete gene list extracted from PANTHER and corresponding to all identified proteins**. Click here for file
