# Supplementary material for: A large-scale electrophoresis- and chromatography-based determination of gene expression profiles in bovine brain capillary endothelial cells after the re-induction of blood-brain barrier properties
Source: Proteome Sci. 2010 Nov 15;8:57. doi: 10.1186/1477-5956-8-57 (PMC2993662; doi:10.1186/1477-5956-8-57)
Supplement: Additional File 1 — Table S1. File reporting the complete gene list identified from the 2D-PAGE experiment. [file 1477-5956-8-57-S1.PDF]

Table 1 - An alphabetical, name-based list of all proteins identified in the 2-DE approach

Spot number	Protein name	Identity [a]		PMF Mascot Score [b]	Seq. pI [c]	Seq. MW [d] [kDa]	Total Seq. Cov. [%] [e]	Pep. Count [f]	Combined MS/MS Mascot Score [g]
		NCBI	UniProt	NCBI / Uniprot					
158	14-3-3 protein beta/alpha (Protein kinase C inhibitor protein 1) (KCIP-1)	gi|71153774	**1433B_BOVIN**	129 / 154	4,6	27,9	53,5		
150	14-3-3 protein epsilon (14-3-3E)	gi|67464424	1433E_SHEEP	163 / 152	4,5	29,2	58		
156	14-3-3 protein zeta/delta (Protein kinase C inhibitor protein 1) (KCIP-1) (Factor activating exoenzyme S) (FA	gi|83754467	1433Z_HUMAN	135 / 128	4,8	29,2	48,4		
157	14-3-3 protein zeta/delta (Protein kinase C inhibitor protein 1) (KCIP-1) (Factor activating exoenzyme S) (FA	gi|83754467	1433Z_HUMAN	177 / 142	4,6	27,7	53,5		
55	26S protease regulatory subunit 6B (Proteasome 26S subunit ATPase 4) (MIP224) (MB67-interacting protei	gi|89064750	**PRS6B_BOVIN**	248 / 246	5	47,3	62,2		
82	26S protease regulatory subunit 7 (Proteasome 26S subunit ATPase 2)	gi|4506209	**PRS7_BOVIN**	311 / 376	5,6	48,5	72,2		
110 *	26S proteasome non-ATPase regulatory subunit 13	--	**PSD13_BOVIN**	-- / 81	5,5	42,9	26,1		
107	40S ribosomal protein SA	gi|146674809	**RSSA_BOVIN**	130 / 158	4,7	32,7	43,9	3	132,3
215	40S ribosomal protein SA	gi|146674809	**RSSA_BOVIN**	132 / 114	5,1	28,8	52,5		
153	40S ribosomal protein SA	gi|161761214	**RSSA_BOVIN**	143 / 160	4,6	32,7	59,2	4	169,4
99	40S ribosomal protein SA (p40) (34/67 kDa laminin receptor)	gi|149018243	**RSSA_BOVIN**	131 / 114	9,2	27,9	64,2	2	86,1
132	60S acidic ribosomal protein P0	gi|2293577	**RLA0_BOVIN**	224 / 279	5,1	32,4	56,3		
163	6-phosphogluconolactonase	gi|84370199	**6PGL_BOVIN**	117 / 126	5,5	27,5	48,4	4	115,3
199	A Chain A, Cdc42ACK GTPASE-Binding Domain Complex	gi|5542168	**CDC42_BOVIN**	98 / 113	5,4	20,4	56		
12	A Chain A, Crystal Structure Of Bovine Hsc70(Aa1-554)e213aD214A MUTANT	gi|78101017	**HSP7C_BOVIN**	217 / 247	6	60,9	49,3		
96	A Chain A, The Crystal Structure Of The Exon Junction Complex At 3.2 A Resolution	gi|496902	**IF4A3_BOVIN**	91 / 113	6,3	46,7	34,4		
200	Abhydrolase domain containing 14B	gi|157428006	**ABHEB_BOVIN**	100 / 108	5,6	22,6	26,2	3	177,8
126	Acidic ribosomal phosphoprotein PO	gi|2293577	**RLA0_BOVIN**	261 / 297	5,1	32,4	67,9	3	184,3
208	Actin related protein 2/3 complex, subunit 5, 16kDa	gi|78369476	**ARPC5_BOVIN**	84 / 102	5,8	16,3	51,7		
65	Actin, cytoplasmic 1 (Beta-actin)	gi|109492380	ACTB_CAMDR	217 / 220	5,2	41,8	66,7	4	242,5
79	Actin, cytoplasmic 1 (Beta-actin)	gi|109492380	ACTB_CANFA	107 / 105	6	41,6	32,3	2	96,7
80	Actin, cytoplasmic 1 (Beta-actin)	gi|109492380	**ACTB_BOVIN**	170 / 160	6	41,6	32,3	2	85
81	Actin, cytoplasmic 1 (Beta-actin)	gi|194376310	ACTB_CERPY	109 / 115	5,5	40,4	34,6	2	68,2
89	Actin, cytoplasmic 1 (Beta-actin)	gi|14250401	**ACTB_BOVIN**	117 / 133	6	41,6	31,7	1	70,6
90	Actin, cytoplasmic 1 (Beta-actin)	gi|62897625	ACTB_CANFA	99 / 101	6	41,6	39,7	1	60,9
100	Actin, cytoplasmic 1 (Beta-actin)	gi|15277603	ACTB_CANFA	172 / 208	6	41,6	37,3	3	203,8
101	Actin, cytoplasmic 1 (Beta-actin)	gi|15277603	ACTB_CANFA	127 / 128	6	41,6	42,1	1	31,9
102	Actin, cytoplasmic 1 (Beta-actin)	gi|148744172	**ACTB_BOVIN**	247 / 253	6	41,6	45,6	2	192,6
103	Actin, cytoplasmic 1 (Beta-actin)	gi|15277503	ACTB_CANFA	187 / 220	6	41,6	45,6	3	226,2
111	Actin, cytoplasmic 1 (Beta-actin)	gi|49868	**ACTB_BOVIN**	236 / 215	5,2	41,8	63,2	3	231,5
115	Actin, cytoplasmic 1 (Beta-actin)	gi|49868	**ACTB_BOVIN**	166 / 201	5,2	41,8	48,8	2	137
135	Actin, cytoplasmic 1 (Beta-actin)	gi|60389477	**ACTB_BOVIN**	121 / 160	5,2	41,8	35,7	2	106,5
1	Actinin, alpha 4	gi|148238040	**ACTN4_BOVIN**	376 / 441	5,2	104,9	61,4		
2	Actinin, alpha 4 isoform 14 [PREDICTED]	gi|73947744	**ACTN4_BOVIN**	289 / 327	5,2	108,3	44,4		
71	Actin-like protein 3 (Actin-related protein 3) (Actin-2)	gi|161728791	**ARP3_BOVIN**	294 / 345	5,5	47,2	63,3	1	70,1
155	Actin-related protein 2/3 complex subunit 2 (ARP2/3 complex 34 kDa subunit) (p34-ARC)	gi|77736371	**ARPC2_BOVIN**	226 / 268	7	34,3	70	2	118,9
211	Actin-related protein 2/3 complex subunit 5-like protein	gi|77736419	**ARP5L_BOVIN**	96 / 128	6,2	16,9	46,4		
212	ADP-ribosylation factor 1	gi|40889633	ARF1_RAT	204 / 228	6,4	20,6	88,3	2	99,9
61	AL7A1_BOVIN Alpha-aminoadipic semialdehyde dehydrogenase	gi|109940193	**AL7A1_BOVIN**	123 / 165	5,6	55,3	38,9		
62	AL7A1_BOVIN Alpha-aminoadipic semialdehyde dehydrogenase	gi|109940193	**AL7A1_BOVIN**	140 / 158	5,6	55,3	48,7		
72	Aldehyde dehydrogenase 9 family, member A1	gi|114051782	**AL9A1_BOVIN**	160 / 191	5,8	53,9	47,4		
127 *	Aldose reductase (EC 1.1.1.21) (AR) (Aldehyde reductase) (20-alpha-hydroxysteroid dehydrogenase)		**ALDR_BOVIN**	-- / 96	5,7	35,9	46	2	38,4
73	Alpha-enolase (EC 4.2.1.11)	gi|87196501	**ENOA_BOVIN**	103 / 113	6,4	47,2	29,1		
85	Alpha-enolase (EC 4.2.1.11) (2-phospho-D-glycerate hydro-lyase) (Non-neural enolase)	gi|87196501	**ENOA_BOVIN**	179 / 213	6,4	47,2	54,3		
88	Alpha-enolase (EC 4.2.1.11) (2-phospho-D-glycerate hydro-lyase) (Non-neural enolase)	gi|87196501	**ENOA_BOVIN**	262 / 289	6,4	47,2	68,8	2	117,9
129	Annexin A1 (Annexin I) (Lipocortin I) (Calpactin II) (Chromobindin-9) (p35)	gi|61553085	**ANXA1_BOVIN**	289 / 272	6,4	38,9	71,4	3	269,3
139	Annexin A3 (Annexin III)	gi|78369184	**ANXA3_BOVIN**	354 / 360	6,6	36	78,6	3	131,5
148	Annexin A4 (Annexin IV) (Lipocortin IV) (Endonexin I) (Chromobindin-4)	gi|48374083	**ANXA4_BOVIN**	219 / 278	5,4	35,7	63,2	1	33,1
133	Annexin A5 (Annexin V) (Lipocortin V) (Endonexin II) (Calphobindin I)	gi|109932864	**ANXA5_BOVIN**	313 / 377	4,7	35,9	83,1	1	66,1
134	Annexin A5 (Annexin V) (Lipocortin V) (Endonexin II) (Calphobindin I)	gi|109932864	**ANXA5_BOVIN**	234 / 282	4,7	35,9	77,2		
143	Annexin A5 (Annexin V) (Lipocortin V) (Endonexin II) (Calphobindin I)	gi|120474983	**ANXA5_BOVIN**	329 / 287	4,7	35,9	66,6	2	58,4

[a] Bovine origine of proteins referenced in Uniprot (to default in NCBInr) is highlighted in bold
[b] Mascot score obtained from the peptide mass fingerprint (scores correspond to the mesure of certainty, p-value<0.05)
[c] Isoelectric point of listed proteins
[d] Molecular Weight of listed proteins
[e] Total sequence coverage corresponding to the peptide mass fingerprint
[f] Peptide count corresponds to the number of MS-fragmented peptides
[g] Combined score corresponding to the sum of all individual peptide frangmentation fingerprint scores

Table 1 : (continued)

Spot number	Protein name	Identity [a] NCBI	Identity [a] UniProt	PMF Mascot Score [b] NCBI / Uniprot	Seq. pI [c]	Seq. MW [d] [kDa]	Total Seq. Cov. [%] [e]	Pep. Count [f]	Combined MS/MS Mascot Score [g]
183	Apolipoprotein A-I precursor (Apo-AI) (ApoA-I)	gi|245563	**APOA1_BOVIN**	312 / 366	5,6	30,3	67,9		
184	Apolipoprotein A-I precursor (Apo-AI) (ApoA-I)	gi|245563	**APOA1_BOVIN**	276 / 272	5,6	30,3	60		
52	ATP synthase subunit beta, mitochondrial precursor (EC 3.6.3.14)	gi|3660251	**ATPB_BOVIN**	266 / 238	5	56,2	63,3	4	313,7
95	Calcium-binding mitochondrial carrier protein SCaMC-1	gi|149642721	**SCMC1_BOVIN**	133 / 161	7,6	53,3	42,1		
173	Calpain, small subunit 1	gi|27806277	**CPNS1_BOVIN**	159 / 168	4,9	27,9	69,6	2	78,6
27	Calreticulin precursor (CRP55) (Calregulin) (HACBP)	gi|146186828	**CALR_BOVIN**	299 / 288	4,2	48	48,9	2	96,4
28	Calreticulin precursor (CRP55) (Calregulin) (HACBP)	gi|146186828	**CALR_BOVIN**	298 / 286	4,2	48	54,2	1	59,2
124	Capping protein (actin filament) muscle Z-line, alpha 2	gi|61316470	**CAZA2_BOVIN**	158 / 185	5,5	32,8	60,7		
112	Cathepsin D precursor (EC 3.4.23.5)	gi|13637914	**CATD_BOVIN**	187 / 216	6,7	42,5	51,5		
167	Cathepsin D precursor (EC 3.4.23.5)	gi|13637914	**CATD_BOVIN**	187 / 209	6,7	42,5	48,7		
206	Chain A, Bovine Cytochrome B(5)	gi|1372997	**CYB5_BOVIN**	193 / 198	4,9	11,2	85,7	2	171,1
31	Chaperonin containing TCP1, subunit 5 (epsilon) [PREDICTED]	**gi|194676636**	TCPE_RAT	126 / 111	5,5	59,6	26,2		
30	Chaperonin isoform 4 [PREDICTED]	**gi|74353880**	CH60_RAT	148 / 169	8,9	50,6	37		
162	Chloride intracellular channel protein 4 (Intracellular chloride ion channel protein p64H1)	gi|122692293	**CLIC4_BOVIN**	159 / 189	5,5	28,6	71,8		
166	Chloride intracellular channel protein 4 (Intracellular chloride ion channel protein p64H1)	gi|122692293	**CLIC4_BOVIN**	210 / 288	5,5	28,6	82,1		
36	Coatomer subunit delta (Delta-coat protein) (Delta-COP) (Archain)	gi|76635416	**COPD_BOVIN**	115 / 132	5,8	57,2	39,1		
113	Cystathionine gamma-lyase	gi|66792918	**CGL_BOVIN**	95 / 78	6	44,4	43		
69	Cytochrome b-c1 complex subunit 1, mitochondrial precursor	gi|4139392	**QCR1_BOVIN**	170 / 193	5,4	49,2	51,6		
59	Cytosol aminopeptidase (EC 3.4.11.1) (Leucine aminopeptidase) (Proline aminopeptidase)	gi|157830427	**AMPL_BOVIN**	232 / 238	5,6	52,8	56,1		
37	Dihydropyrimidinase-related protein 2 (DRP-2) (Neural-specific protein NSP60)	gi|115496400	**DPYL2_BOVIN**	176 / 217	5,9	62,2	50,2		
42	Dihydropyrimidinase-related protein 2 (DRP-2) (Neural-specific protein NSP60)	gi|115496400	**DPYL2_BOVIN**	123 / 173	5,9	62,2	41,6		
53	Dynactin 2 (p50)	gi|77736063	**DCTN2_BOVIN**	209 / 239	4,9	44,3	49,4		
54	EIF3F_HUMAN	**gi|194673284**	EIF3F_HUMAN	217 / 184	0	0	0	2	140,7
164	Endoplasmic reticulum protein ERp29 (Fragments)	gi|115495555	**ERP29_BOVIN**	148 / 185	5,5	29	45	4	103,8
141	Eukaryotic translation elongation factor 1 beta 2-like	gi|58760396	**EF1B_BOVIN**	131 / 128	4,3	24,6	33		
86	Eukaryotic translation elongation factor 1 gamma	gi|95769122	**EF1G_BOVIN**	116 / 173	6,3	50,2	26,9		
97	Eukaryotic translation elongation factor 1 gamma	gi|95769122	**EF1G_BOVIN**	107 /101	6,3	50,2	38,5	2	37,1
207	Eukaryotic translation initiation factor 5A-1 (eIF-5A-1) (eIF-5A1)	gi|148680528	IF5A1_RABIT	86 / 75	4,9	16,7	43,1	2	55,6
26	Ezrin (p81) (Cytovillin) (Villin-2)	gi|27806351	**EZRI_BOVIN**	177 / 231	6	68,6	39,3		
147	F-actin capping protein subunit beta (CapZ beta)	gi|83649737	CAPZB_MOUSE	165 / 173	5,4	31,2	50,4		
60	Fascin homolog 1, actin-bundling protein	**gi|78045491**	FSCN1_HUMAN	146 / 86	6,6	54,8	56,4		
118	Galactokinase	gi|150247075	**GALK1_BOVIN**	205 /219	5,6	42,3	66,3		
114	Gelsolin-like capping protein	gi|30466254	**CAPG_BOVIN**	153 / 179	6,2	38,9	43,6	3	140,8
149	Glyoxalase domain containing 4	**gi|156120555**	GLOD4_HUMAN	163 / 65	5,6	33,2	46,6		
185	Growth factor receptor-bound protein 2 isoform 1	gi|4504111	GRB2_RAT	197 / 205	6,4	23,5	74,4		
109	Guanine nucleotide-binding protein G(i), alpha-2 subunit (Adenylate cyclase-inhibiting G alpha protein)	**gi|198282135**	GNAI2_HUMAN	221 / 202	5,2	40,3	48,3	1	30
16	Heat shock 70 kDa protein 1A (HSP70.1)	gi|56757663	**HS71B_BOVIN**	220 / 283	5,6	70,2	50,9		
5	Heat shock 70kDa protein 5	gi|115495027	**GRP78_BOVIN**	333 / 131	4,9	72,4	53,7	3	161,7
9	Heat shock cognate 71 kDa protein (Heat shock 70 kDa protein 8)	gi|78101017	**HSP7C_BOVIN**	297 / 296	5,2	70,9	51,7	3	145,6
10	Heat shock cognate 71 kDa protein (Heat shock 70 kDa protein 8)	gi|78101017	**HSP7C_BOVIN**	269 / 290	5,4	71,2	46	3	109
11	Heat shock cognate 71 kDa protein (Heat shock 70 kDa protein 8)	gi|78101017	**HSP7C_BOVIN**	232 / 258	6,2	50,4	48,4	4	203,3
180	Heat-shock protein beta-1 (HspB1) (Heat shock 27 kDa protein) (HSP 27)	gi|85542053	**HSPB1_BOVIN**	218 / 225	6	22,4	78,6	3	244,2
121	Heterogeneous nuclear ribonucleoprotein A/B (hnRNP A/B) (CArG-binding factor-A)	**gi|94966849**	ROAA_MOUSE	96.1 / 70	8,7	30,6	36,1		
98	Heterogeneous nuclear ribonucleoprotein H (hnRNP H)	gi|73970377	HNRH1_HUMAN	71 / 82	5,9	49,1	37,9		
186	Heterogeneous nuclear ribonucleoprotein H (hnRNP H)	gi|73970353	HNRH1_MOUSE	168 / 144	5,9	49	35,5	2	85,8
214	Heterogeneous nuclear ribonucleoprotein K	gi|77736071	**HNRPK_BOVIN**	168 / 231	5	51	32,8		
93	Heterogeneous nuclear ribonucleoprotein K (hnRNP K)	gi|77736071	**HNRPK_BOVIN**	63 / 84	5	51	26,3		
136	Heterogeneous nuclear ribonucleoprotein K (hnRNP K)	gi|77736071	**HNRPK_BOVIN**	189 / 232	5	51	38,4	1	98,8
175	Homeobox prox 1	**gi|78369460**	CN166_PONAB	210 / 235	6,2	28,1	70,1		
120	Isocitrate dehydrogenase [NAD] subunit alpha, mitochondrial precursor (Fragment)	gi|1182011	**IDH3A_BOVIN**	111 / 130	6	37,6	30,3	2	63,7
94	Isocitrate dehydrogenase [NADP] cytoplasmic (EC 1.1.1.42)	gi|75832090	**IDHC_BOVIN**	182 / 194	6,1	46,8	53,4		
19	Lysyl-tRNA synthetase	**gi|77735669**	SYK_MOUSE	90 / 87.2	6,3	71,3	19,4		

[a] Bovine origine of proteins referenced in Uniprot (to default in NCBInr) is highlighted in bold
[b] Mascot score obtained from the peptide mass fingerprint (scores correspond to the mesure of certainty, p-value<0.05)
[c] Isoelectric point of listed proteins
[d] Molecular Weight of listed proteins
[e] Total sequence coverage corresponding to the peptide mass fingerprint
[f] Peptide count corresponds to the number of MS-fragmented peptides
[g] Combined score corresponding to the sum of all individual peptide frangmentation fingerprint scores

Table 1 : (continued)

Spot number	Protein name	Identity [a]		PMF Mascot Score [b]	Seq. pI [c]	Seq. MW [d] [kDa]	Total Seq. Cov. [%] [e]	Pep. Count [f]	Combined MS/MS Mascot Score [g]
		NCBI	UniProt	NCBI / Uniprot					
113	Mannose-1-phosphate guanyltransferase beta	gi|114053237	**GMPPB_BOVIN**	88 / 103	6,2	39	31,7		
147	Microtubule-associated protein RP/EB family member 1 (APC-binding protein EB1)	gi|115496618	**MARE1_BOVIN**	126 / 123	5,1	30	53,2		
6	Moesin	gi|149042267	**MOES_BOVIN**	166 / 161	9,2	45,4	48,4		
18	Moesin	gi|114050715	**MOES_BOVIN**	202 / 276	5,8	67,9	40		
20	Moesin	gi|114050715	**MOES_BOVIN**	168 / 206	5,8	67,9	43,3	2	46,9
21	Moesin	gi|114050715	**MOES_BOVIN**	76 / 95.6	5,8	67,9	28,9		
22	Moesin	gi|114050715	**MOES_BOVIN**	206 / 251	5,8	67,9	47,5		
7	NADH dehydrogenase (ubiquinone) Fe-S protein 1	gi|27807355	**NDUS1_BOVIN**	226 / 285	5,8	79,4	47,3		
121	NADH dehydrogenase [ubiquinone] 1 alpha subcomplex subunit 10, mitochondrial precursor	Gi|28603782	**NDUAA_BOVIN**	71 / 78.8	6,6	39,2	42,6		
92	NADH:ubiquinone oxidoreductase	gi|833783	**NDUS2_BOVIN**	112 / 107	5,8	48,9	39,5		
67 *	Nestin [PREDICTED]	**gi|76612380**		120 / –	4,2	175,5	13,4		
123	Nestin [PREDICTED]	**gi|76612380**	NEST_HUMAN	157 / 87.2	4,3	176,6	8,9	3	137,5
4	Neutral alpha-glucosidase AB	gi|151553519	GANAB_PIG	272 / 94.5	5,6	106,6	20,9		
116	NG,NG-dimethylarginine dimethylaminohydrolase 1 (Dimethylarginine dimethylaminohydrolase 1)	gi|109157318	**DDAH1_BOVIN**	273 / 306	5,6	31,1	65,8		
117	NG,NG-dimethylarginine dimethylaminohydrolase 1 (Dimethylarginine dimethylaminohydrolase 1)	gi|109157318	**DDAH1_BOVIN**	85 / 114	5,6	31,1	47,9		
161	Osteoclast-stimulating factor 1	gi|27806039	**OSTF1_BOVIN**	137 / 162	5,2	23,8	69,2		
187	Parkinson disease (autosomal recessive, early onset) 7	gi|62751849	**PARK7_BOVIN**	158 / 170	7,7	20	68,8		
191	Peroxiredoxin 2	gi|27807469	**PRDX2_BOVIN**	127 / 140	5,3	21,9	56,8	3	231,8
192	Peroxiredoxin 3	gi|27806083	**PRDX3_BOVIN**	113 / 144	7,9	28,2	54,1	3	140,1
174	Peroxiredoxin-6 (EC 1.11.1.15) (Antioxidant protein 2) (1-Cys peroxiredoxin)	gi|27807167	**PRDX6_BOVIN**	214 / 232	6	24,9	65,9	1	92,3
140 *	Phosphatidylinositol transfer protein alpha isoform (PtdIns transfer protein alpha)	--	**PIPNA_BOVIN**	-- / 72	6,1	31,8	28,3		
176	Phosphoglycerate mutase 1 (brain)	gi|77404217	**PGAM1_BOVIN**	220 / 242	6,8	28,8	66,9		
57	Plastin 3	gi|114052248	**PLST_BOVIN**	167 / 223	5,3	71,8	36,8		
51	Plastin 3 isoform 3 [PREDICTED]	gi|74008150	**PLST_BOVIN**	99 / 122	5,4	70,1	28,7		
8	Procollagen-lysine, 2-oxoglutarate 5-dioxygenase 3 isoform 6 [PREDICTED]	gi|194678667	PLOD3_PONAB	261 / 175	5,6	84,7	27,9		
40 *	Procollagen-proline, 2-oxoglutarate 4-dioxygenase (proline 4-hydroxylase), alpha polypeptide II	**gi|74353841**		87 / --	5,6	55,5	25,9		
87	Proliferation-associated protein 2G4 (Proliferation-associated protein 1) (Protein p38-2G4)	gi|109097235	PA2G4_MOUSE	148 / 151	6,4	43,5	49,4		
41	Prolyl 4-hydroxylase alpha-2 subunit precursor	**gi|226874885**	P4HA2_MOUSE	253 / 174	5,5	61	35,2	2	62,4
170	Proteasome (prosome, macropain) subunit, beta type 7	gi|149047897	PSB7_RAT	64 / 72	9,1	29,9	17,3		
179	Proteasome (prosome, macropain) subunit, beta type, 4	gi|77735487	**PSB4_BOVIN**	122 / 140	5,4	29	53,8		
165	Proteasome activator complex subunit 1 (Proteasome activator 28-alpha subunit)	gi|91680535	**PSME1_BOVIN**	231 / 246	5,7	28,6	77,1		
145	Proteasome activator complex subunit 3 (Proteasome activator 28-gamma subunit)	gi|6755214	PSME3_PIG	227 / 283	5,6	29,5	66,5	1	38,9
154	Proteasome subunit alpha type 1 (Proteasome component C2) (Macropain subunit C2)	gi|149719471	PSA1_RAT	89 / 122	6,2	29,6	48,1		
197	Proteasome subunit beta type 9 precursor	gi|77735723	**PSB9_BOVIN**	137 / 181	4,9	23,4	56,2	1	30,9
42	Protein disulfide-isomerase A3 precursor (EC 5.3.4.1) (Disulfide isomerase ER-60)	gi|729433	**PDIA3_BOVIN**	187 / 215	6,2	56,9	43,8		
43	Protein disulfide-isomerase A3 precursor (EC 5.3.4.1) (Disulfide isomerase ER-60)	gi|729433	**PDIA3_BOVIN**	271 / 259	6,2	56,9	55,6	3	209,9
44	Protein disulfide-isomerase A3 precursor (EC 5.3.4.1) (Disulfide isomerase ER-60)	gi|729433	**PDIA3_BOVIN**	250 / 289	6,2	56,9	46,5	1	60,7
168	Purine nucleoside phosphorylase (EC 2.4.2.1) (Inosine phosphorylase) (PNP)	gi|1042206	**PNPH_BOVIN**	244 / 255	5,9	32,1	65,7	3	211,8
169	Purine nucleoside phosphorylase (EC 2.4.2.1) (Inosine phosphorylase) (PNP)	gi|163310884	**PNPH_BOVIN**	102 / 125	5,9	32,1	50,5		
122	Pyrophosphatase (inorganic)	gi|115495033	**IPYR_BOVIN**	133 / 133	5,1	32,8	42,6		
83	Rab GDP dissociation inhibitor beta (Rab GDI beta) (Guanosine diphosphate dissociation inhibitor 2)	gi|76253900	**GDIB_BOVIN**	277 / 330	5,9	50,5	67,4	2	53,7
84	Rab GDP dissociation inhibitor beta (Rab GDI beta) (Guanosine diphosphate dissociation inhibitor 2)	gi|76253900	**GDIB_BOVIN**	315 / 365	5,9	50,5	77,1	3	103,1
24	Radixin	gi|115496125	**RADI_BOVIN**	221 / 206	6	68,5	38,9		
25	Radixin	gi|115496125	**RADI_BOVIN**	189 / 225	6	68,5	43,1		
213	Ran-specific GTPase-activating protein (Ran-binding protein 1)	gi|77736119	**RANG_BOVIN**	90 / 99	4,8	23,7	35,4	1	42,2
142	Ras-related protein Rab-11B	gi|14249144	**RB11B_BOVIN**	139 / 162	5,6	24,3	47		
75 *	Reticulocalbin 1, EF-hand calcium binding domain	**gi|157073966**	RCN1_HUMAN	123 / 62	4,6	38,7	39,9	2	90
46	Retinoblastoma-binding protein mRbAp48	gi|1016275	**RBBP4_BOVIN**	164 / 191	4,8	51,7	38,8		
160	Rho GDP dissociation inhibitor (GDI) alpha	gi|28603774	**GDIR1_BOVIN**	171 / 184	5	23,4	74		
137	Spermidine synthase (EC 2.5.1.16) (Putrescine aminopropyltransferase) (SPDSY)	**gi|76637216**	SPEE_HUMAN	82 / 86.5	5,2	33,8	22,5		
14	Stress-70 protein, mitochondrial precursor (75 kDa glucose-regulated protein)	gi|114601963	**GRP75_BOVIN**	248 / 251	5,8	73,6	44,5	4	181,4
70	RuvB-like protein 2	gi|84370077	**RUVB2_BOVIN**	188 / 236	5,4	50,9	42		

[a] Bovine origine of proteins referenced in Uniprot (to default in NCBInr) is highlighted in bold
[b] Mascot score obtained from the peptide mass fingerprint (scores correspond to the mesure of certainty, p-value<0.05)
[c] Isoelectric point of listed proteins
[d] Molecular Weight of listed proteins
[e] Total sequence coverage corresponding to the peptide mass fingerprint
[f] Peptide count corresponds to the number of MS-fragmented peptides
[g] Combined score corresponding to the sum of all individual peptide frangmentation fingerprint scores

Table 1 : (continued)

Spot number	Protein name	Identity [a]		PMF Mascot Score [b]	Seq. pI [c]	Seq. MW [d] [kDa]	Total Seq. Cov. [%] [e]	Pep. Count [f]	Combined MS/MS Mascot Score [g]
		NCBI	UniProt	NCBI / Uniprot					
15	Stress-70 protein, mitochondrial precursor (75 kDa glucose-regulated protein)	gi|77735995	**GRP75_BOVIN**	298 / 312	5,8	73,6	51,4	4	224,9
35	Stress-induced-phosphoprotein 1	gi|78369310	**STIP1_BOVIN**	232 / 274	6	62,4	58,6		
91	Succinyl-CoA ligase [ADP-forming] beta-chain, mitochondrial precursor [PREDICTED]	gi|115496292	**SUCB1_BOVIN**	116 / 172	6,7	47,5	49,7		
90	Succinyl-CoA ligase [GDP-forming] beta-chain, mitochondrial precursor [PREDICTED]	gi|146231894	**SUCB2_BOVIN**	133 / 102	8,7	46,6	44,9	1	49,8
34	T-complex protein 1 subunit alpha (TCP-1-alpha) (CCT-alpha)	gi|84000143	**TCPA_BOVIN**	205 / 242	5,7	60,2	38,1		
60	T-complex protein 1 subunit beta (TCP-1-beta) (CCT-beta)	gi|77735435	**TCPB_BOVIN**	197 / 160	6,2	57,3	48,5		
33 *	T-complex protein 1 subunit epsilon	**--**	TCPE_RAT	-- / 85	5,7	59,6	15		
38	T-complex protein 1 subunit gamma (TCP-1-gamma) (CCT-gamma)	gi|164448698	**TCPG_BOVIN**	171 / 206	6,4	60,5	50,3		
32	TCPE_RAT	**gi|194676636**	TCPE_RAT	116 / 83.5	5,5	59,6	23,5		
63	Thioredoxin domain containing 4 (endoplasmic reticulum)	gi|78042524	**ERP44_BOVIN**	280 / 293	5	46,8	52,2	1	41,2
56 *	Thioredoxin domain-containing protein 5 precursor (Thioredoxin-like protein p46)	**gi|119915902**	TXND5_HUMAN	81.4 / 58	5,6	47,6	13,2		
64	Thioredoxin domain-containing protein 5 precursor (Thioredoxin-like protein p46) [PREDICTED]	**gi|119915902**	TXND5_HUMAN	141 / 85	5,3	45,8	46,2		
45	Thioredoxin reductase 1, cytoplasmic (EC 1.8.1.9) (TR) (TR1)	gi|27807129	**TRXR1_BOVIN**	145 / 203	6,1	54,7	45,9		
130	Thioredoxin-like protein 1 (Thioredoxin-related protein)	gi|109122274	TXNL1_RAT	170 / 176	4,7	32,1	63,2		
23	TNF receptor-associated protein 1	gi|84579841	**TRAP1_BOVIN**	143 / 149	6,7	79,3	32,4		
210	Transgelin-2	gi|61888874	**TAGL2_BOVIN**	234 / 262	9,4	22,3	80,3		
3	Transitional endoplasmic reticulum ATPase (TER ATPase) (Valosin-containing protein)	gi|73971230	**TERA_BOVIN**	85 / 99	5,1	90,7	24,9		
131	Translational elongation factor 1 delta	gi|146231746	**EF1D_BOVIN**	95 / 116	5,3	30,8	53,8		
177	Translationally-controlled tumor protein (TCTP)	gi|62177164	**TCTP_BOVIN**	143 / 153	4,7	19,6	61	2	106,1
209	Translocon-associated protein subunit delta precursor (TRAP-delta)	gi|84370035	**SSRD_BOVIN**	154 / 150	5,4	18,8	44,2	2	114,1
181	Triosephosphate isomerase (EC 5.3.1.1) (TIM) (Triose-phosphate isomerase)	gi|61888856	**TPIS_BOVIN**	273 / 296	6,5	26,5	90,7	1	45,3
50	Tryptophanyl-tRNA synthetase, cytoplasmic	gi|110283011	**SYWC_BOVIN**	218 / 225	5,7	52,1	48,8	3	178,3
66	Tubulin beta-5 chain	gi|74141821	**TBB5_BOVIN**	248 / 248	4,7	49,7	59,9		
68	Tubulin beta-5 chain	gi|7106439	**TBB5_BOVIN**	301 / 328	4,6	49,7	66,4	2	66,3
146	Tubulin beta-5 chain	gi|16198437	**TBB5_BOVIN**	194 / 185	4,6	49,6	32,4	1	36,1
201	UMP-CMP kinase (Uridine monophosphate/cytidine monophosphate kinase)	gi|150383501	**KCY_BOVIN**	97 / 118	6	22,3	28,6		
138 *	Uridine phosphorylase 1	**gi|149773558**	--	133/ --	5,5	33,9	56,3		
29	Vimentin	gi|110347570	**VIME_BOVIN**	233 / 245	5,1	53,5	51,2		39,7
47	Vimentin	gi|110347570	**VIME_BOVIN**	352 / 346	5,1	53,5	76,6	2	133,9
48	Vimentin	gi|110347570	**VIME_BOVIN**	315 / 369	5,1	53,5	63,9	2	93,2
49	Vimentin	gi|110347570	**VIME_BOVIN**	272 / 308	5,1	53,5	66		
76	Vimentin	gi|110347570	**VIME_BOVIN**	326 / 371	5,1	53,5	64,1	2	101,5
77	Vimentin	gi|145226795	**VIME_BOVIN**	394 / 408	5,1	53,5	67,5	2	101,7
105	Vimentin	gi|110347570	**VIME_BOVIN**	287 / 285	5,1	53,5	62,8		
106	Vimentin	gi|110347570	**VIME_BOVIN**	236 / 262	5,1	53,5	59,8		
144	Vimentin	gi|110347570	**VIME_BOVIN**	99 / 112	5,1	53,5	28		
151	Vimentin	gi|110347570	**VIME_BOVIN**	202 / 205	5,1	53,5	36,6		
152	Vimentin	gi|110347570	**VIME_BOVIN**	263 / 264	5,1	53,5	49,9	4	278,5
159	Vimentin	gi|110347570	**VIME_BOVIN**	157 / 156	5,1	53,5	31,6		
171	Vimentin	gi|110347570	**VIME_BOVIN**	156 / 176	5,1	53,5	36,6		
172	Vimentin	gi|110347570	**VIME_BOVIN**	144 / 174	5,1	53,5	30,8	2	105,1
182	Vimentin	gi|289450	**VIME_BOVIN**	101 / 97	4,9	53,6	26,9		
188	Vimentin	gi|21431723	**VIME_BOVIN**	102 / 110	6,6	31	42,5		
189	Vimentin	gi|110347570	**VIME_BOVIN**	149 / 163	4,9	53,5	34,8	1	60,5
190	Vimentin	gi|21431723	**VIME_BOVIN**	69 / 81	5,1	53,5	20		
193	Vimentin	gi|110347570	**VIME_BOVIN**	181 / 192	4,9	53,5	43,2		
194	Vimentin	gi|110347570	**VIME_BOVIN**	118 / 137	4,9	53,5	32,7		
195	Vimentin	gi|75075845	**VIME_BOVIN**	143 / 146	4,9	53,5	37,6		
202	Vimentin	gi|207656	**VIME_BOVIN**	114 / 94.4	5,1	53,5	18,7		
203	Vimentin	gi|110347570	**VIME_BOVIN**	137 / 152	5,1	53,5	30,1	1	73,1
205	Vimentin	gi|110347570	**VIME_BOVIN**	124 / 138	5,1	53,5	35,3		
39	Vimentin	gi|110347570	**VIME_BOVIN**	372 / 369	5,1	53,5	74,6	2	123,2
78	Vimentin	gi|145226795	**VIME_BOVIN**	176 / 202	4,9	53,5	51		
204	Vimentin	gi|110347570	**VIME_BOVIN**	169 / 181	4,8	51,5	43,3		
196	Vimentin (Fragments)	gi|21431723	VIME_PIG	87 / 88	6,6	31	41,8		
104	Vimentin variant 3	gi|110347570	**VIME_BOVIN**	108 / 113	4,9	53,5	34,2		
13	V-type proton ATPase catalytic subunit A	gi|74002620	**VATA_BOVIN**	208 / 187	5,1	69	40,9	1	45,3

[a] Bovine origine of proteins referenced in Uniprot (to default in NCBInr) is highlighted in bold
[b] Mascot score obtained from the peptide mass fingerprint (scores correspond to the mesure of certainty, p-value<0.05)
[c] Isoelectric point of listed proteins
[d] Molecular Weight of listed proteins
[e] Total sequence coverage corresponding to the peptide mass fingerprint
[f] Peptide count corresponds to the number of MS-fragmented peptides
[g] Combined score corresponding to the sum of all individual peptide frangmentation fingerprint scores
