# Supplementary material for: A large-scale electrophoresis- and chromatography-based determination of gene expression profiles in bovine brain capillary endothelial cells after the re-induction of blood-brain barrier properties
Source: Proteome Sci. 2010 Nov 15;8:57. doi: 10.1186/1477-5956-8-57 (PMC2993662; doi:10.1186/1477-5956-8-57)
Supplement: Additional File 2 — Table S2. File corresponding to the complete gene list identified from the LC-MS experiment. [file 1477-5956-8-57-S2.PDF]

Table 2 - An alphabetical, name-based list of all proteins identified by LC-MS approach

Accession	Gene symbol	Protein name	Theo. pI[a]	Theo. MW [kDa][b]	Seq.Cov.[c]	Peptide Count[d]	Combined Mascot-Score[e]
U5S1_BOVIN	EFTUD2	116 kDa U5 small nuclear ribonucleoprotein component	4,9	109,3	9,0	2	41,5
1433B_BOVIN	YWHAB	14-3-3 protein beta/alpha (Protein kinase C inhibitor protein 1) (KCIP-1)	4,6	27,9	11,8	3	259,5
1433E_BOVIN	YWHAE	14-3-3 protein epsilon (14-3-3E)	4,5	29,2	22,4	5	367,1
1433Z_BOVIN	YWHAZ	14-3-3 protein zeta/delta (Protein kinase C inhibitor protein 1) (KCIP-1)	4,6	27,7	11,8	3	187,4
PRS6B_BOVIN	PSMC4	26S protease regulatory subunit 6B	5,1	47,3	7,0	1	95,3
PSD13_BOVIN	PSMD13	26S proteasome non-ATPase regulatory subunit 13	5,4	42,8	9,8	2	216
PSDE_HUMAN	PSMD14	26S proteasome non-ATPase regulatory subunit 14	6,1	34,6	7,1	1	107,4
PSMD2_BOVIN	PSMD2	26S proteasome non-ATPase regulatory subunit 2	5,1	100,2	13,0	4	229
PSMD6_BOVIN	PSMD6	26S proteasome non-ATPase regulatory subunit 6	5,5	45,5	15,0	1	51,5
RS10_BOVIN	RPS10	40S ribosomal protein S10	10,2	18,9	23,0	2	90,5
RS13_BOVIN	RPS13	40S ribosomal protein S13	11,0	17,1	10,0	1	43,4
RS15_BOVIN	RPS15	40S ribosomal protein S15	10,8	16,9	15,3	1	128,6
RS16_HUMAN	RPS16	40S ribosomal protein S16	10,8	16,3	15,9	2	67,1
RS18_BOVIN	RPS18	40S ribosomal protein S18	11,5	17,6	5,3	1	61,3
RS24_BOVIN	RPS24	40S ribosomal protein S24	11,4	15,2	9,2	1	82,3
RS3_MOUSE	RPS3	40S ribosomal protein S3	10,3	26,7	15,6	2	87,4
RS3A_MOUSE	RPS3A	40S ribosomal protein S3a	10,4	29,7	6,5	1	66,5
RS5_BOVIN	Rps5	40S ribosomal protein S5	10,4	22,7	13,8	1	59,3
RS8_HUMAN	RPS8	40S ribosomal protein S8	11,0	24,1	13,5	2	113,5
RS9_BOVIN	RPS9	40S ribosomal protein S9	10,7	22,6	5,0	1	71,4
RSSA_BOVIN	RPSA	40S ribosomal protein SA (p40) (Protein C10)	4,6	32,7	9,5	3	184
AL9A1_BOVIN	ALDH9A1	4-trimethylaminobutyraldehyde dehydrogenase (EC 1.2.1.47)	5,8	53,9	4,5	1	56,5
CH60_BOVIN	HSPD1	60 kDa heat shock protein, mitochondrial (Hsp60) (60 kDa chaperonin) (Fragment)	5,6	61,1	21,0	4	288,1
RLA1_BOVIN	RPLP1	60S acidic ribosomal protein P1	4,3	11,5	14,0	1	38
RL12_BOVIN	RPL12	60S ribosomal protein L12	10,3	17,8	9,1	1	70,5
RL3_BOVIN	RPL3	60S ribosomal protein L3	10,8	45,9	8,7	2	38,6
RL4_BOVIN	RPL4	60S ribosomal protein L4	11,7	47,3	9,0	2	199,2
RL6_BOVIN	RPL6	60S ribosomal protein L6	11,2	32,5	11,2	2	143,1
RL7_BOVIN	RPL7	60S ribosomal protein L7	11,2	29,2	7,7	1	40,9
RL9_BOVIN	RPL9	60S ribosomal protein L9	10,5	21,9	11,5	1	48,4
K6PL_BOVIN	PFKL	6-phosphofructokinase, liver type	7,0	85,2	9,0	2	41,6
6PGD_SHEEP	PGD	6-phosphogluconate dehydrogenase, decarboxylating (EC 1.1.1.44)	8,7	52,8	11,8	3	326,3
GRP78_BOVIN	HSPA5	78 kDa glucose-regulated protein	5,1	72,4	17,0	8	461,9
AN32A_BOVIN	ANP32A	Acidic leucine-rich nuclear phosphoprotein 32 family member A	3,8	28,6	5,2	1	61,3
ACTC_BOVIN	ACTC1	Actin, alpha cardiac muscle 1	5,2	42,0	45,0	11	703,2
ACTA_BOVIN	ACTA2	Actin, aortic smooth muscle (Alpha-actin-2)	5,1	42,0	43,5	11	664,6
ACTG_BOVIN	ACTG1	Actin, cytoplasmic 2 (Gamma-actin)	5,2	41,8	45,9	14	1254,6
ARP3_BOVIN	ACTR3	Actin-like protein 3 (Actin-related protein 3) (Actin-2)	5,5	47,2	7,7	2	119,7
ARP2_BOVIN	ACTR2	Actin-related protein 2	6,3	44,7	26,0	3	135,6
ARPC2_BOVIN	ARPC2	Actin-related protein 2/3 complex subunit 2 (ARP2/3 complex 34 kDa subunit)	7,0	34,3	14,7	3	219,8
ARPC4_BOVIN	ARPC4	Actin-related protein 2/3 complex subunit 4	8,5	19,7	4,0	1	36,1
ACBP_BOVIN	DBI	Acyl-CoA-binding protein (ACBP) (Diazepam-binding inhibitor) (DBI) (Endozepine) (EP)	6,2	9,9	20,9	1	54,6
APT_BOVIN	APRT	Adenine phosphoribosyltransferase (EC 2.4.2.7) (APRT)	9,1	19,5	7,2	1	66,1
ADA_BOVIN	ADA	Adenosine deaminase (EC 3.5.4.4) (Adenosine aminohydrolase)	5,2	40,8	14,4	2	89,6
SAHH_BOVIN	AHCY	Adenosylhomocysteinase	5,9	47,6	8,0	2	35
CAP1_BOVIN	CAP1	Adenylyl cyclase-associated protein 1	7,2	51,2	14,0	3	299,9
ADIPO_MOUSE	Adipoq	Adiponectin precursor (Adipocyte, C1q and collagen domain-containing protein)	5,2	26,8	6,1	1	41,4

[a] Isoelectric point of listed proteins
[b] Molecular Weight of listed proteins
[c] Total sequence coverage corresponding to the peptide mass fingerprint
[d] Peptide count corresponds to the number of MS-fragmented peptides
[e] Combined score corresponding to the sum of all individual peptide frangmentation fingerprint scores

Table 2 - (Continued)

Accession	Gene symbol	Protein name	Theo. pI[a]	Theo. MW [kDa][b]	Seq.Cov.[c]	Peptide Count[d]	Combined Mascot-Score[e]
ADT3_BOVIN	SLC25A6	ADP/ATP translocase 3 (Adenine nucleotide translocator 2)	10,4	32,7	9,8	3	133,8
ARF1_BOVIN	ARF1	ADP-ribosylation factor 1	6,4	20,6	10,0	1	102
ALDH2_BOVIN	ALDH2	Aldehyde dehydrogenase, mitochondrial precursor (EC 1.2.1.3)	8,6	56,7	12,7	5	206,9
ACTN1_BOVIN	ACTN1	Alpha-actinin-1	5,3	102,9	23,0	11	768,4
ACTN4_BOVIN	ACTN4	Alpha-actinin-4	5,3	104,9	33,0	18	1187,9
ACTZ_RAT	Actr1a	Alpha-centractin	6,2	42,6	17,0	3	114,7
ENOA_BOVIN	ENO1	Alpha-enolase (EC 4.2.1.11) (2-phospho-D-glycerate hydro-lyase) (Non-neural enolase)	6,4	47,2	29,1	8	686,2
ACY1_MOUSE	Acy1	Aminoacylase-1	5,9	45,8	7,0	1	31,2
ANFY1_HUMAN	ANKFY1	Ankyrin repeat and FYVE domain-containing protein 1 (Ankyrin repeats hooked to a zinc finger motif)	5,7	128,3	2,7	1	53,5
ANXA1_BOVIN	ANXA1	Annexin A1 (Annexin I) (Lipocortin I) (Phospholipase A2 inhibitory protein)	6,4	38,9	18,8	5	426
ANX11_BOVIN	ANXA11	Annexin A11	7,5	54,0	5,4	1	45,8
ANXA2_BOVIN	ANXA2	Annexin A2 (Annexin II) (Lipocortin II) (Calpactin I heavy chain)	7,6	38,5	27,8	8	608,2
ANXA3_BOVIN	ANXA3	Annexin A3 (Annexin III)	6,6	36,0	14,6	3	238,5
ANXA4_BOVIN	ANXA4	Annexin A4 (Annexin IV) (Lipocortin IV) (Endonexin I)	5,4	35,7	13,5	2	140,1
ANXA5_BOVIN	ANXA5	Annexin A5 (Annexin V) (Lipocortin V) (Endonexin II)	4,7	35,9	39,4	8	533,1
ANXA6_BOVIN	ANXA6	Annexin A6 (Annexin VI) (Lipocortin VI) (Fragment)	5,3	69,8	2,4	2	52,6
AP1B1_MOUSE	AP2B1	AP-1 complex subunit beta-1 (Adapter-related protein complex 1 beta-1 subunit) (Clathrin assembly protein complex 1 beta)	4,9	103,9	5,0	2	117,9
AP2A2_BOVIN	AP2A2	AP-2 complex subunit alpha-2	6,5	103,8	6,0	2	46,7
AP2B1_BOVIN	AP2B1	AP-2 complex subunit beta	5,2	104,5	8,0	3	97,4
AP2M1_BOVIN	AP2M1	AP-2 complex subunit mu OS=Bos taurus	9,6	49,6	10,0	2	55,4
APOA1_BOVIN	APOA1	Apolipoprotein A-I precursor (Apo-AI) (ApoA-I)	5,6	30,3	3,4	1	40,8
APOA2_BOVIN	APOA2	Apolipoprotein A-II precursor (Apo-AII) (ApoA-II) (Antimicrobial peptide BAMP-1)	9,1	11,2	16,0	1	32,2
SYNC_BOVIN	NARS	Asparaginyl-tRNA synthetase, cytoplasmic	6,3	64,4	17,0	5	140,7
SYDC_BOVIN	DARS	Aspartyl-tRNA synthetase, cytoplasmic	6,4	57,0	11,0	1	35,6
PEA15_MOUSE	PEA15	Astrocytic phosphoprotein PEA-15	4,8	15,0	25,4	2	85,4
ATLA3_HUMAN	ATL3	Atlastin-3	5,4	60,5	11,0	1	43,4
AT5F1_BOVIN	ATP5F1	ATP synthase B chain, mitochondrial precursor (EC 3.6.3.14)	9,8	28,8	9,0	2	37,1
ATP5H_BOVIN	ATP5H	ATP synthase D chain, mitochondrial (EC 3.6.3.14)	5,9	18,5	8,1	1	99,2
ATPK_BOVIN	ATP5J2	ATP synthase f chain, mitochondrial (EC 3.6.3.14)	10,3	10,2	27,6	2	105,5
ATPG_BOVIN	ATP5C1	ATP synthase gamma chain, mitochondrial precursor (EC 3.6.3.14)	9,8	33,1	10,1	1	41,4
ATPO_BOVIN	ATP5O	ATP synthase O subunit, mitochondrial precursor (EC 3.6.3.14)	10,5	23,3	15,5	2	79,1
ATPA_BOVIN	ATP5A1	ATP synthase subunit alpha, mitochondrial	9,2	59,7	24,0	4	493,2
ATPB_BOVIN	ATP5B	ATP synthase subunit beta, mitochondrial precursor (EC 3.6.3.14)	5,0	56,2	47,9	15	1102,5
ATP5J_BOVIN	ATP5J	ATP synthase-coupling factor 6, mitochondrial	9,3	12,5	17,0	1	40,4
ATAD3_BOVIN	ATAD3	ATPase family AAA domain-containing protein 3	9,3	66,1	4,0	1	30,5
ATAD5_HUMAN	ATAD5	ATPase family AAA domain-containing protein 5	9,3	207,4	4,0	2	34,3
ACLY_BOVIN	ACLY	ATP-citrate synthase	6,8	119,7	6,0	1	61,1
DHX9_BOVIN	DHX9	ATP-dependent RNA helicase A (EC 3.6.1.-) (Nuclear DNA helicase II)	6,5	141,9	4,9	4	161,5
DDX3X_HUMAN	DDX3X	ATP-dependent RNA helicase DDX3X (EC 3.6.1.-) (DEAD box protein 3, X-chromosomal)	6,8	73,1	7,0	3	153,3
BGAL_BOVIN	GLB1	Beta-galactosidase	7,0	73,4	12,0	2	81,8
SYEP_HUMAN	EPRS	Bifunctional aminoacyl-tRNA synthetase [Includes: Glutamyl-tRNA synthetase (EC 6.1.1.17)	8,6	162,9	1,6	3	127,7
PUR9_BOVIN	ATIC	Bifunctional purine biosynthesis protein PURH	6,4	64,4	12,0	4	159,3
CAD13_BOVIN	CDH13	Cadherin-13 precursor	4,7	78,1	7,7	3	137,1
SCMC1_BOVIN	SLC25A24	Calcium-binding mitochondrial carrier protein	6,9	53,3	7,0	1	42,2
CALM_BOVIN	CALM	Calmodulin (CaM)	3,9	16,7	10,8	1	114,4
CALX_MOUSE	Canx	Calnexin precursor	4,3	67,2	5,8	2	187,5

[a] Isoelectric point of listed proteins
[b] Molecular Weight of listed proteins
[c] Total sequence coverage corresponding to the peptide mass fingerprint
[d] Peptide count corresponds to the number of MS-fragmented peptides
[e] Combined score corresponding to the sum of all individual peptide frangmentation fingerprint scores

Table 2 - (Continued)

Accession	Gene symbol	Protein name	Theo. pI[a]	Theo. MW [kDa][b]	Seq.Cov.[c]	Peptide Count[d]	Combined Mascot-Score[e]
CPNS1_BOVIN	CAPNS1	Calpain small subunit 1	5,1	27,9	22,0	2	53,7
CAN2_BOVIN	CAPN2	Calpain-2 catalytic subunit (EC 3.4.22.53) (Calpain-2 large subunit) (Fragment)	4,3	23,9	10,6	2	129,3
CALR_BOVIN	CALR	Calreticulin precursor (CRP55) (Calregulin) (HACBP)	4,2	48,0	12,2	3	226,8
CALU_BOVIN	CALU	Calumenin precursor	4,3	37,1	10,5	2	40,1
KAP2_BOVIN	PRKAR2A	cAMP-dependent protein kinase type II-alpha regulatory subunit	4,6	44,9	18,5	4	178,6
CTNB1_BOVIN	CTNNB1	Catenin beta-1	5,5	85,5	12,0	1	110,9
CATB_BOVIN	CTSB	Cathepsin B	5,7	36,6	5,0	1	117,7
CATD_BOVIN	CTSD	Cathepsin D precursor (EC 3.4.23.5)	6,7	42,5	5,1	2	121,8
CAV1_BOVIN	CAV1	Caveolin-1	5,6	20,6	7,9	1	82,6
CDC42_BOVIN	CDC42	Cell division control protein 42 homolog	6,2	21,2	15,0	2	98
CLIC1_BOVIN	CLIC1	Chloride intracellular channel protein 1	5,0	26,8	15,0	2	150,5
CLIC2_HUMAN	CLIC2	Chloride intracellular channel protein 2 (XAP121)	5,3	28,3	5,3	1	30,7
DYH5_MOUSE	Dnah5	Ciliary dynein heavy chain 5 (Axonemal beta dynein heavy chain 5) (Mdnah5)	5,8	527,2	0,8	2	32,5
CISY_BOVIN	CS	Citrate synthase, mitochondrial precursor (EC 2.3.3.1)	8,9	51,7	4,9	2	63
CLH1_BOVIN	CLTC	Clathrin heavy chain 1	5,4	191,5	20,1	20	1316,7
COTL1_BOVIN	COTL1	Coactosin-like protein	4,9	15,9	11,3	1	114,4
COPB_BOVIN	COPB1	Coatomer subunit beta	5,7	107,1	9,0	3	79,2
COPG_BOVIN	COPG	Coatomer subunit gamma (Gamma-coat protein) (Gamma-COP)	5,2	97,3	3,7	2	54
COF1_BOVIN	CFL1	Cofilin-1 (Cofilin, non-muscle isoform)	9,2	18,4	6,7	1	75,4
CO1A1_MOUSE	Col1a1	Collagen alpha-1(I) chain precursor	5,6	137,9	1,5	1	45,9
CPNE1_HUMAN	CPNE1	Copine-1 (Copine I)	5,4	59,0	5,4	2	121,5
COLI_BALBO	POMC	Corticotropin-lipotropin (Pro-opiomelanocortin) (POMC) [Contains: Corticotropin (Fragment)	9,3	4,5	56,4	1	37,1
CAND1_BOVIN	CAND1	Cullin-associated NEDD8-dissociated protein 1	5,5	136,3	5,0	3	197,1
CYTB_BOVIN	CSTB	Cystatin B (Stefin B)	6,4	11,1	12,2	1	48,2
CRIP2_BOVIN	CRIP2	Cysteine-rich protein 2	9,0	22,6	15,0	1	90,3
CX6A1_BOVIN	COX6A1	Cytochrome c oxidase polypeptide VIa-liver (EC 1.9.3.1) (SSG)	6,2	9,5	21,2	1	39
ACOC_BOVIN	ACO1	Cytoplasmic aconitate hydratase	8,1	98,1	9,0	1	33,8
DYHC1_MOUSE	Dync1h1	Cytoplasmic dynein 1 heavy chain 1	6,0	531,7	8,0	14	649,7
DC1I2_BOVIN	Dync1i2	Cytoplasmic dynein 1 intermediate chain 2	5,2	68,4	7,0	1	31,1
DOCK8_HUMAN	DOCK8	Dedicator of cytokinesis protein 8	6,4	230,7	1,0	1	33
AL4A1_BOVIN	ALDH4A1	Delta-1-pyrroline-5-carboxylate dehydrogenase, mitochondrial	8,4	61,5	9,0	1	57,1
DHPR_BOVIN	QDPR	Dihydropteridine reductase	6,9	25,5	14,0	1	30
DPYL2_BOVIN	DPYSL2	Dihydropyrimidinase-related protein 2 (DRP-2) (Neural-specific protein NSP60)	5,9	62,2	19,4	5	358,4
CATC_BOVIN	CTSC	Dipeptidyl-peptidase 1 precursor (EC 3.4.14.1) (Dipeptidyl-peptidase I)	8,5	51,9	8,9	3	239,9
OST48_BOVIN	DDOST	Dolichyl-diphosphooligosaccharide--protein glycosyltransferase 48 kDa subunit	5,5	48,8	7,0	1	88,8
RPN1_PIG	RPN1	Dolichyl-diphosphooligosaccharide--protein glycosyltransferase subunit 1	6,1	68,7	6,0	2	68,2
RPN2_BOVIN	RPN2	Dolichyl-diphosphooligosaccharide--protein glycosyltransferase subunit 2	5,5	69,2	18,0	4	212,8
STT3A_BOVIN	STT3A	Dolichyl-diphosphooligosaccharide--protein glycosyltransferase subunit STT3A	8,3	80,6	7,0	1	93,3
DYSF_BOVIN	DYSF	Dysferlin	5,4	239,2	7,0	3	62,3
ENTP8_HUMAN	ENTPD8	Ectonucleoside triphosphate diphosphohydrolase 8	5,2	53,9	--	2	34,6
EHD2_MOUSE	EHD2	EH domain-containing protein 2	6,1	61,1	12,0	4	354,1
EHD4_MOUSE	Ehd4	EH-domain-containing protein 4 (mPAST2)	6,3	61,4	4,1	2	34,8
ETFB_BOVIN	ETFB	Electron transfer flavoprotein subunit beta (Beta-ETF)	9,3	27,6	16,1	2	52,8
EF1A1_BOVIN	EEF1A1	Elongation factor 1-alpha 1 (EF-1-alpha-1) (Elongation factor 1 A-1) (eEF1A-1)	9,7	50,1	10,2	3	134,9
EF1G_BOVIN	EEF1G	Elongation factor 1-gamma	6,2	50,3	18,0	4	232,5
EF2_BOVIN	EEF2	Elongation factor 2 (EF-2)	6,4	95,2	18,2	9	700,2

[a] Isoelectric point of listed proteins
[b] Molecular Weight of listed proteins
[c] Total sequence coverage corresponding to the peptide mass fingerprint
[d] Peptide count corresponds to the number of MS-fragmented peptides
[e] Combined score corresponding to the sum of all individual peptide frangmentation fingerprint scores

Table 2 - (Continued)

Accession	Gene symbol	Protein name	Theo. pI[a]	Theo. MW [kDa][b]	Seq.Cov.[c]	Peptide Count[d]	Combined Mascot-Score[e]
ENPL_BOVIN	HSP90B1	Endoplasmin precursor (Heat shock protein 90 kDa beta member 1) (94 kDa glucose-regulated protein)	4,6	92,4	11,3	7	447,4
IF4A1_HUMAN	EIF4A1	Eukaryotic initiation factor 4A-I (EC 3.6.1.-) (ATP-dependent RNA helicase eIF4A-1)	5,2	46,1	15,5	4	232,7
EIF3E_BOVIN	EIF3E	Eukaryotic translation initiation factor 3 subunit E	5,7	52,2	9,0	2	100,9
EIF3M_BOVIN	EIF3M	Eukaryotic translation initiation factor 3 subunit M	5,4	42,4	10,0	1	132,1
IF5A1_BOVIN	EIF5A	Eukaryotic translation initiation factor 5A-1 (eIF-5A-1) (eIF-5A1)	4,9	16,7	15,7	2	159,8
XPO1_MOUSE	XPO1	Exportin-1	5,7	123,0	4,0	1	38,3
ESYT1_MOUSE	ESYT1	Extended synaptotagmin-1	5,6	121,5	7,0	1	62,2
CAPZB_BOVIN	CAPZB	F-actin capping protein subunit beta (CapZ beta)	6,0	33,7	4,7	1	56,5
CAZA1_BOVIN	CAPZA1	F-actin-capping protein subunit alpha-1	5,5	32,9	6,0	1	48,5
FSCN1_HUMAN	FSCN1	Fascin (Singed-like protein) (55 kDa actin-bundling protein) (p55)	7,0	54,4	12,6	4	335,3
FAS_BOVIN	FASN	Fatty acid synthase (EC 2.3.1.85)	6,2	274,4	0,9	1	47,1
FERM2_MOUSE	Fermt2	Fermitin family homolog 2	6,3	77,8	7,0	1	35,4
FLNA_HUMAN	FLNA	Filamin-A (Alpha-filamin) (Filamin-1) (Endothelial actin-binding protein) (Actin-binding protein 280) (ABP-280)	5,7	280,4	7,6	11	550,2
FLNB_HUMAN	FLNB	Filamin-B (FLN-B) (Beta-filamin) (Actin-binding-like protein) (Thyroid autoantigen)	5,4	278,0	3,5	6	259
FKB1A_BOVIN	FKBP1A	FK506-binding protein 1A (EC 5.2.1.8) (Peptidyl-prolyl cis-trans isomerase) (PPIase) (Immunophilin FKBP12)	9,2	11,8	13,1	1	80,7
FXR1_BOVIN	FXR1	Fragile X mental retardation syndrome-related protein 1	5,8	69,8	8,0	1	50,4
ALDOA_RAT	Aldoa	Fructose-bisphosphate aldolase A (EC 4.1.2.13) (Muscle-type aldolase)	9,3	39,2	8,8	2	156
GALK1_BOVIN	GALK1	Galactokinase	5,8	42,2	9,0	2	121,2
LEG9_BOVIN	LGALS9	Galectin-9	9,4	39,3	9,0	1	51,6
GCN1L_HUMAN	GCN1L1	GCN1-like protein 1 (HsGCN1)	7,4	292,4	0,9	2	46
USO1_BOVIN	USO1	General vesicular transport factor p115	4,8	107,4	5,0	2	100,9
GFPT2_BOVIN	GFPT2	Glucosamine--fructose-6-phosphate aminotransferase [isomerizing] 2	7,6	77,0	7,0	1	110,8
G6PI_BOVIN	GPI	Glucose-6-phosphate isomerase (EC 5.3.1.9) (GPI) (Phosphoglucose isomerase) (PGI)	8,0	62,7	2,7	1	74,8
GLU2B_BOVIN	PRKCSH	Glucosidase 2 subunit beta precursor (Glucosidase II subunit beta)	4,2	60,1	3,6	1	86,7
DHE3_BOVIN	GLUD1	Glutamate dehydrogenase 1, mitochondrial precursor (EC 1.4.1.3) (GDH)	7,8	61,5	5,7	2	70,3
SYQ_BOVIN	QARS	Glutaminyl-tRNA synthetase (EC 6.1.1.18) (Glutamine--tRNA ligase) (GlnRS)	6,5	87,6	1,9	1	45
GPX1_BOVIN	GPX1	Glutathione peroxidase 1 (EC 1.11.1.9) (GSHPx-1) (GPx-1) (Cellular glutathione peroxidase)	7,7	22,6	4,4	1	38,6
GSTP1_BOVIN	GSTP1	Glutathione S-transferase P (EC 2.5.1.18) (GST class-pi)	7,8	23,5	34,0	4	372,6
G3P_BOVIN	GAPDH	Glyceraldehyde-3-phosphate dehydrogenase (EC 1.2.1.12) (GAPDH)	9,3	35,7	28,6	7	667,9
SYG_MOUSE	Gars	Glycyl-tRNA synthetase (EC 6.1.1.14) (Glycine--tRNA ligase) (GlyRS)	6,2	81,8	1,8	1	33,4
RAN_CANFA	RAN	GTP-binding nuclear protein Ran (GTPase Ran) (Ras-like protein TC4)	7,8	24,3	6,5	1	36,4
GNAI2_HUMAN	GNAI2	Guanine nucleotide-binding protein G(i), alpha-2 subunit (Adenylate cyclase-inhibiting G alpha protein)	5,2	40,3	13,0	3	173
GNAS2_BOVIN	GNAS	Guanine nucleotide-binding protein G(s) subunit alpha isoforms short	5,5	45,7	0,0	1	62,7
HS71A_BOVIN	HSPA1A	Heat shock 70 kDa protein 1A	5,7	70,2	25,0	5	206,3
HSP74_HUMAN	HSPA4	Heat shock 70 kDa protein 4 (Heat shock 70-related protein APG-2) (HSP70RY)	5,0	94,2	4,0	1	72,6
HSP7C_BOVIN	HSPA8	Heat shock cognate 71 kDa protein (Heat shock 70 kDa protein 8)	5,4	71,2	31,1	11	787,6
HS90A_BOVIN	HSP90AA1	Heat shock protein HSP 90-alpha	4,8	84,5	10,7	6	414,4
HS90B_BOVIN	HSP90AB1	Heat shock protein HSP 90-beta	4,8	83,1	18,1	9	491,3
HSPB1_BOVIN	HSPB1	Heat-shock protein beta-1 (HspB1) (Heat shock 27 kDa protein) (HSP 27)	6,0	22,4	18,4	3	93,5
HDGF_BOVIN	HDGF	Hepatoma-derived growth factor (HDGF)	4,6	26,6	21,3	3	140,9
ROA1_BOVIN	HNRNPA1	Heterogeneous nuclear ribonucleoprotein A1 (Helix-destabilizing protein) (Fragment)	8,9	22,1	16,9	3	128
ROA3_MOUSE	HNRNPA3	Heterogeneous nuclear ribonucleoprotein A3 (hnRNP A3)	9,6	39,6	4,2	1	31,8
HNRH1_MOUSE	HNRNPH1	Heterogeneous nuclear ribonucleoprotein H (hnRNP H)	5,9	49,0	13,4	3	214,6
HNRPK_BOVIN	HNRNPK	Heterogeneous nuclear ribonucleoprotein K (hnRNP K)	5,0	51,0	6,5	2	98,5
HNRPL_MOUSE	Hnrnpl	Heterogeneous nuclear ribonucleoprotein L (hnRNP L)	6,7	60,1	4,7	1	52,6
HNRPQ_HUMAN	SYNCRIP	Heterogeneous nuclear ribonucleoprotein Q (hnRNP Q) (hnRNP-Q) (Synaptotagmin-binding, cytoplasmic RNA-interacting protein)	9,1	69,6	2,9	1	50,8
HNRPR_HUMAN	HNRNPR	Heterogeneous nuclear ribonucleoprotein R (hnRNP R)	8,8	70,9	2,2	1	46,8

[a] Isoelectric point of listed proteins
[b] Molecular Weight of listed proteins
[c] Total sequence coverage corresponding to the peptide mass fingerprint
[d] Peptide count corresponds to the number of MS-fragmented peptides
[e] Combined score corresponding to the sum of all individual peptide frangmentation fingerprint scores

Table 2 - (Continued)

Accession	Gene symbol	Protein name	Theo. pI[a]	Theo. MW [kDa][b]	Seq.Cov.[c]	Peptide Count[d]	Combined Mascot-Score[e]
HNRPU_MOUSE	HNRNPU	Heterogeneous nuclear ribonucleoprotein U	5,9	87,9	6,0	2	102,9
HNRL2_MOUSE	HNRNPUL2	Heterogeneous nuclear ribonucleoprotein U-like protein 2	4,8	85,0	6,0	1	84,1
ROA2_BOVIN	HNRNPA2B1	Heterogeneous nuclear ribonucleoproteins A2/B1	8,7	36,0	26,0	3	236,4
HINT1_BOVIN	HINT1	Histidine triad nucleotide-binding protein 1	6,3	13,8	30,0	1	58
H2A2C_BOVIN	HIST2H2AC	Histone H2A type 2-C	10,9	14,0	49,0	5	217,6
H2B1K_BOVIN	HIST1H2BK	Histone H2B type 1-K	10,8	13,7	12,0	3	126
H4_MOUSE	Hist1h4a	Histone H4	11,8	11,2	29,4	3	147
CDC37_BOVIN	CDC37	Hsp90 co-chaperone Cdc37	5,1	44,6	4,0	1	57,1
HYOU1_MOUSE	HYOU1	Hypoxia up-regulated protein 1	5,1	111,1	2,7	2	65,5
IMB1_MOUSE	KPNB1	Importin beta-1 subunit (Karyopherin beta-1 subunit) (Nuclear factor P97)	4,5	97,1	5,4	3	145,1
IPO5_MOUSE	Ipo5	Importin-5	4,8	123,5	5,0	1	48,8
ITB1_BOVIN	ITGB1	Integrin beta-1 precursor (Fibronectin receptor subunit beta) (Integrin VLA-4 subunit beta) (CD29 antigen)	5,1	88,2	2,4	1	96,4
IDH3A_BOVIN	IDH3A	Isocitrate dehydrogenase [NAD] subunit alpha, mitochondrial precursor (EC 1.1.1.41)	6,9	39,6	7,4	2	169,2
SYIC_MOUSE	IARS	Isoleucyl-tRNA synthetase, cytoplasmic (EC 6.1.1.5) (Isoleucine--tRNA ligase) (IleRS) (IRS)	6,1	144,2	1,7	2	54,9
PLAK_BOVIN	JUP	Junction plakoglobin	5,8	81,8	9,0	1	46,3
LGUL_MOUSE	GLO1	Lactoylglutathione lyase (EC 4.4.1.5) (Methylglyoxalase) (Aldoketomutase) (Glyoxalase I) (Glx I)	5,1	20,7	5,5	1	30,9
LAP2B_MOUSE	Tmpo	Lamina-associated polypeptide 2 isoforms beta/delta/epsilon/gamma	9,9	50,1	3,1	1	67,8
SYLC_MOUSE	LARS	Leucyl-tRNA synthetase, cytoplasmic (EC 6.1.1.4) (Leucine--tRNA ligase) (LeuRS)	6,7	134,1	2,2	1	72,5
ILEU_BOVIN	SERPINB1	Leukocyte elastase inhibitor	5,7	42,2	14,0	2	96,2
LDHA_BOVIN	LDHA	L-lactate dehydrogenase A chain (EC 1.1.1.27) (LDH-A) (LDH muscle subunit) (LDH-M)	9,2	36,4	15,1	3	148,5
MIF_BOVIN	MIF	Macrophage migration inhibitory factor	7,7	12,3	9,0	1	47,6
MVP_BOVIN	MVP	Major vault protein (MVP)	5,3	98,9	4,5	2	103,1
MDHM_BOVIN	MDH2	Malate dehydrogenase, mitochondrial	8,8	35,6	13,0	3	115,3
PGRC1_BOVIN	PGRMC1	Membrane-associated progesterone receptor component 1	4,6	21,6	12,0	1	80,7
PGRC2_MOUSE	Pgrmc2	Membrane-associated progesterone receptor component 2 (Fragment)	4,9	23,0	6,5	1	96,2
CNNM4_HUMAN	CNNM4	Metal transporter CNNM4	5,8	86,6	0,0	1	31
MAP1S_BOVIN	MAP1S	Microtubule-associated protein 1S	6,2	112,4	3,0	1	40,6
MTCH2_BOVIN	MTCH2	Mitochondrial carrier homolog 2	8,4	33,3	14,0	1	100,8
TOM40_BOVIN	TOMM40	Mitochondrial import receptor subunit TOM40 homolog	6,8	37,7	4,0	1	43,3
MPPA_BOVIN	PMPCA	Mitochondrial-processing peptidase subunit alpha	6,6	58,1	5,0	1	65,3
MOES_BOVIN	MSN	Moesin	5,9	67,9	30,0	13	1046,8
MYL6_BOVIN	MYL6	Myosin light polypeptide 6 (Myosin light chain alkali 3) (Myosin light chain 3) (MLC-3) (LC17)	4,4	16,8	26,0	4	166,2
MYL9_BOVIN	MYL9	Myosin regulatory light polypeptide 9	4,7	19,9	5,0	1	32,2
MYH9_CANFA	MYH9	Myosin-9	5,5	226,3	13,0	11	917,6
MYO1C_BOVIN	MYO1C	Myosin-Ic	9,3	121,9	9,0	3	107
NB5R3_BOVIN	CYB5R3	NADH-cytochrome b5 reductase 3	6,9	34,0	24,0	3	145
NEST_HUMAN	NES	Nestin	4,3	176,6	4,0	1	55,4
GANAB_PIG	GANAB	Neutral alpha-glucosidase AB precursor (EC 3.2.1.84) (Glucosidase II subunit alpha)	5,6	106,6	4,7	2	52,1
DDAH1_BOVIN	DDAH1	NG,NG-dimethylarginine dimethylaminohydrolase 1 (EC 3.5.3.18) (Dimethylargininase-1)	5,6	31,1	13,0	2	86,1
NIBL1_MOUSE	FAM129B	Niban-like protein 1	5,7	84,8	9,0	2	68,4
NONO_MOUSE	NONO	Non-POU domain-containing octamer-binding protein (NonO protein)	9,6	54,5	7,8	2	196,3
NOLC1_RAT	NOLC1	Nucleolar and coiled-body phosphoprotein 1	9,6	73,5	2,9	1	34,5
NOP56_BOVIN	NOP56	Nucleolar protein Nop56 (Nucleolar protein 5A)	9,9	66,3	4,2	1	44,1
NPM_BOVIN	NPM1	Nucleophosmin	4,6	32,7	11,0	2	160,7
NDKB_BOVIN	NME2	Nucleoside diphosphate kinase B (EC 2.7.4.6) (NDK B) (NDP kinase B)	9,0	17,3	25,7	3	106,2

[a] Isoelectric point of listed proteins
[b] Molecular Weight of listed proteins
[c] Total sequence coverage corresponding to the peptide mass fingerprint
[d] Peptide count corresponds to the number of MS-fragmented peptides
[e] Combined score corresponding to the sum of all individual peptide frangmentation fingerprint scores

Table 2 - (Continued)

Accession	Gene symbol	Protein name	Theo. pI[a]	Theo. MW [kDa][b]	Seq.Cov.[c]	Peptide Count[d]	Combined Mascot-Score[e]
NP1L4_BOVIN	NAP1L4	Nucleosome assembly protein 1-like 4	4,6	44,0	13,0	2	92,6
PPIA_BOVIN	PPIA	Peptidyl-prolyl cis-trans isomerase A (EC 5.2.1.8) (PPIase A) (Rotamase A) (Cyclophilin A)	9,5	17,7	11,0	1	142
PRDX2_BOVIN	PRDX2	Peroxiredoxin-2 (EC 1.11.1.15)	5,3	21,9	9,0	2	143,9
MPCP_BOVIN	SLC25A3	Phosphate carrier protein, mitochondrial precursor (PTP) (Solute carrier family 25 member 3)	10,0	40,1	11,6	3	106,5
PEBP1_BOVIN	PEBP1	Phosphatidylethanolamine-binding protein 1 (PEBP-1) (HCNPpp) (Basic cytosolic 21 kDa protein)	7,8	20,8	11,3	1	52,7
PGM1_BOVIN	PGM1	Phosphoglucomutase-1	6,4	61,6	6,0	1	84
PGM2_MOUSE	Pgm2	Phosphoglucomutase-2 (EC 5.4.2.2) (Glucose phosphomutase 2) (PGM 2)	5,7	68,7	1,6	1	47,9
PGK1_BOVIN	PGK1	Phosphoglycerate kinase 1 (EC 2.7.2.3)	9,3	44,4	16,6	4	260,9
PGAM1_BOVIN	PGAM1	Phosphoglycerate mutase 1 (EC 5.4.2.1) (EC 5.4.2.4) (EC 3.1.3.13)	6,8	28,7	34,8	6	324,9
PLST_BOVIN	PLS3	Plastin-3	5,4	70,8	5,0	2	153,8
PECA1_BOVIN	PECAM1	Platelet endothelial cell adhesion molecule precursor (PECAM-1) (CD31 antigen)	7,0	82,5	5,4	2	49,1
PA1B2_BOVIN	PAFAH1B2	Platelet-activating factor acetylhydrolase IB subunit beta (EC 3.1.1.47)	5,5	25,6	12,2	2	95,1
PLEC1_CRIGR	PLEC1	Plectin-1 (PLTN) (PCN) (300 kDa intermediate filament-associated protein) (IFAP300) (Fragment)	5,5	508,7	3,6	8	274,6
PUF60_BOVIN	PUF60	Poly(U)-binding-splicing factor PUF60	5,1	57,1	5,0	1	42
PABP1_BOVIN	PABPC1	Polyadenylate-binding protein 1 (Poly(A)-binding protein 1) (PABP 1)	10,0	70,6	10,2	4	282,5
PTRF_MOUSE	PTRF	Polymerase I and transcript release factor	5,3	43,9	4,6	1	143,8
PTBP1_BOVIN	PTBP1	Polypyrimidine tract-binding protein 1 (PTB)	9,8	57,1	10,2	2	52,7
DDX17_MOUSE	DDX17	Probable ATP-dependent RNA helicase DDX17 (EC 3.6.1.-) (DEAD box protein 17)	9,5	72,4	4,3	2	42,9
DDX5_HUMAN	DDX5	Probable ATP-dependent RNA helicase DDX5 (EC 3.6.1.-) (DEAD box protein 5) (RNA helicase p68)	9,7	69,1	7,8	3	92,1
OSGEP_BOVIN	OSGEP	Probable O-sialoglycoprotein endopeptidase	5,5	36,5	10,0	1	46,7
PROF1_BOVIN	PFN1	Profilin-1 (Profilin I)	9,5	14,9	21,6	2	222,6
PHB2_BOVIN	PHB2	Prohibitin-2	10,2	33,3	4,3	1	58,8
P4HA2_MOUSE	P4ha2	Prolyl 4-hydroxylase alpha-2 subunit precursor (EC 1.14.11.2) (4-PH alpha-2)	5,5	61,0	10,6	3	71,7
PPCE_BOVIN	PREP	Prolyl endopeptidase (EC 3.4.21.26) (Post-proline cleaving enzyme) (PE)	5,5	80,6	4,4	2	65
PTGIS_BOVIN	PTGIS	Prostacyclin synthase (EC 5.3.99.4) (Prostaglandin I2 synthase)	6,8	56,6	8,0	2	104,5
PSME1_BOVIN	PSME1	Proteasome activator complex subunit 1 (Proteasome activator 28-alpha subunit)	5,7	28,6	10,4	2	117,8
PSME2_BOVIN	PSME2	Proteasome activator complex subunit 2 (Proteasome activator 28-beta subunit) (PA28beta) (PA28b)	5,2	27,2	21,4	3	222,9
PSA1_BOVIN	PSMA1	Proteasome subunit alpha type-1	6,2	29,6	9,0	2	40,6
PSA2_BOVIN	PSMA2	Proteasome subunit alpha type-2	6,9	25,9	17,0	1	57,5
PSB1_BOVIN	PSMB1	Proteasome subunit beta type 1 (EC 3.4.25.1)	9,1	26,2	13,3	2	50,1
PSB10_BOVIN	PSMB10	Proteasome subunit beta type 10 precursor (EC 3.4.25.1)	6,0	29,1	7,3	1	45,2
PSB2_BOVIN	PSMB2	Proteasome subunit beta type 2 (EC 3.4.25.1)	6,6	22,9	14,9	2	99
DAPLE_MOUSE	Ccdc88c	Protein Daple	5,8	226,4	6,0	2	36
PDIA3_BOVIN	PDIA3	Protein disulfide-isomerase A3 precursor (EC 5.3.4.1) (Disulfide isomerase ER-60)	6,2	56,9	16,8	8	557,4
PDIA4_BOVIN	PDIA4	Protein disulfide-isomerase A4 precursor (EC 5.3.4.1)	4,8	72,5	5,0	2	86
PDIA6_MESAU	PDIA6	Protein disulfide-isomerase A6 precursor (EC 5.3.4.1) (Protein disulfide isomerase P5)	4,9	48,1	17,3	4	181,1
PDIA1_BOVIN	P4HB	Protein disulfide-isomerase precursor (EC 5.3.4.1) (PDI) (Prolyl 4-hydroxylase subunit beta)	4,7	57,2	17,1	5	433,3
K0284_MOUSE	KIAA0284	Protein KIAA0284	6,4	170,8	2,7	2	33,3
PP1R7_BOVIN	PPP1R7	Protein phosphatase 1 regulatory subunit 7 (Protein phosphatase 1 regulatory subunit 22)	4,7	41,4	5,8	1	49,6
S10A9_HUMAN	S100A9	Protein S100-A9 (S100 calcium-binding protein A9) (Calgranulin-B) (Migration inhibitory factor-related protein 14)	5,7	13,2	24,6	2	119,5
SC23A_BOVIN	SEC23A	Protein transport protein Sec23A	6,8	86,6	10,0	3	241,1
SC24C_HUMAN	SEC24C	Protein transport protein Sec24C	6,7	118,2	6,0	2	82,8
SC31A_HUMAN	SEC31A	Protein transport protein Sec31A	6,4	132,9	2,7	1	51,9
TGM2_BOVIN	TGM2	Protein-glutamine gamma-glutamyltransferase 2 (EC 2.3.2.13) (Tissue transglutaminase)	5,0	77,1	20,2	8	705,3
PNPH_BOVIN	PNP	Purine nucleoside phosphorylase (EC 2.4.2.1) (Inosine phosphorylase) (PNP)	5,9	32,1	47,8	9	783,2
PLBL2_BOVIN	PLBD2	Putative phospholipase B-like 2	8,5	65,7	8,0	1	39,6
PYC_BOVIN	PC	Pyruvate carboxylase, mitochondrial	6,4	129,6	7,0	1	34,6

[a] Isoelectric point of listed proteins
[b] Molecular Weight of listed proteins
[c] Total sequence coverage corresponding to the peptide mass fingerprint
[d] Peptide count corresponds to the number of MS-fragmented peptides
[e] Combined score corresponding to the sum of all individual peptide frangmentation fingerprint scores

Table 2 - (Continued)

Accession	Gene symbol	Protein name	Theo. pI[a]	Theo. MW [kDa][b]	Seq.Cov.[c]	Peptide Count[d]	Combined Mascot-Score[e]
KPYM_HUMAN	PKM2	Pyruvate kinase isozymes M1/M2 (EC 2.7.1.40) (Pyruvate kinase muscle isozyme) (Pyruvate kinase 2/3)	9,0	57,8	18,7	8	646,3
GDIB_BOVIN	GDI2	Rab GDP dissociation inhibitor beta (Rab GDI beta) (Guanosine diphosphate dissociation inhibitor 2) (GDI-2)	5,9	50,5	21,6	7	569,7
RADI_BOVIN	RDX	Radixin	6,0	68,5	11,0	5	373,1
IQGA1_HUMAN	IQGAP1	Ras GTPase-activating-like protein IQGAP1 (p195)	6,1	189,1	3,2	2	157,1
RAIN_HUMAN	RASIP1	Ras-interacting protein 1 (Rain)	9,2	103,4	4,6	2	105,8
RAB10_HUMAN	RAB10	Ras-related protein Rab-10	9,4	22,5	11,0	2	76,5
RAB2A_MOUSE	RAB2A	Ras-related protein Rab-2A	6,1	23,5	6,1	1	32,5
RAB5B_HUMAN	RAB5B	Ras-related protein Rab-5B	9,2	23,7	18,6	3	65,7
RAB5C_BOVIN	RAB5C	Ras-related protein Rab-5C	9,5	23,5	18,5	3	86,5
RAB7A_MOUSE	RAB7A	Ras-related protein Rab-7a	6,4	23,5	19,0	3	82,5
RALB_HUMAN	RALB	Ras-related protein Ral-B	6,3	23,4	15,5	2	89,2
RALB_MOUSE	RALB	Ras-related protein Ral-B	6,3	23,3	15,5	2	90,9
RAP1A_BOVIN	RAP1A	Ras-related protein Rap-1A precursor (GTP-binding protein smg-p21A)	7,2	21,0	6,5	1	68,2
RCN1_MOUSE	RCN1	Reticulocalbin-1 precursor	4,6	38,1	4,9	1	58
ROCK2_MOUSE	ROCK2	Rho-associated protein kinase 2 (EC 2.7.11.1) (Rho-associated, coiled-coil-containing protein kinase 2)	5,7	160,5	2,5	2	46,3
RHOC_BOVIN	RHOC	Rho-related GTP-binding protein RhoC	6,2	22,0	--	1	35
RINI_MOUSE	RNH1	Ribonuclease inhibitor (Ribonuclease/angiogenin inhibitor 1)	4,5	49,8	3,3	1	102,6
UK114_BOVIN	HRSP12	Ribonuclease UK114 (EC 3.1.-.-)	7,0	14,1	19,1	2	55,3
PRPS1_BOVIN	PRPS1	Ribose-phosphate pyrophosphokinase 1	6,5	34,8	22,0	1	39,6
SEPT2_BOVIN	SEPT2	Septin-2	6,2	41,5	4,0	1	60,2
SEPT7_BOVIN	SEPT7	Septin-7 (CDC10 protein homolog)	9,4	50,6	5,3	1	31
2AAA_MOUSE	Ppp2r1a	Serine/threonine-protein phosphatase 2A 65 kDa regulatory subunit A alpha isoform	5,0	65,3	9,0	2	62,4
PP1G_BOVIN	PPP1CC	Serine/threonine-protein phosphatase PP1-gamma catalytic subunit (EC 3.1.3.16)	6,1	37,0	8,7	3	95,1
SERPH_BOVIN	SERPINH1	Serpin H1	9,0	46,5	24,0	6	427,3
ALBU_BOVIN	ALB	Serum albumin precursor (Allergen Bos d 6) (BSA)	5,8	69,2	8,7	4	231,5
ESTD_BOVIN	ESD	S-formylglutathione hydrolase	6,5	31,5	13,0	2	127,5
SPTA2_RAT	Sptan1	Spectrin alpha chain, brain (Spectrin, non-erythroid alpha chain) (Alpha-II spectrin)	5,1	284,5	4,4	6	250,3
SPTB2_HUMAN	SPTBN1	Spectrin beta chain, brain 1 (Spectrin, non-erythroid beta chain 1) (Beta-II spectrin)	5,3	274,5	6,0	10	471,9
UAP56_BOVIN	BAT1	Spliceosome RNA helicase BAT1	5,4	48,9	17,0	4	180,4
SFRS3_BOVIN	SFRS3	Splicing factor, arginine/serine-rich 3	11,6	19,3	14,0	2	105,4
SFPQ_HUMAN	SFPQ	Splicing factor, proline- and glutamine-rich (Polypyrimidine tract-binding protein-associated-splicing factor)	9,9	76,1	8,8	3	186,2
SND1_BOVIN	SND1	Staphylococcal nuclease domain-containing protein 1 (p100 co-activator)	6,8	101,9	4,3	2	77,7
GRP75_BOVIN	HSPA9	Stress-70 protein, mitochondrial	6,0	73,7	8,0	4	174,4
DHSA_BOVIN	SDHA	Succinate dehydrogenase [ubiquinone] flavoprotein subunit, mitochondrial precursor (EC 1.3.5.1)	8,5	72,9	3,9	2	65
SCOT1_HUMAN	OXCT1	Succinyl-CoA:3-ketoacid-coenzyme A transferase 1, mitochondrial	7,1	56,1	7,0	1	40,5
SAE1_BOVIN	SAE1	SUMO-activating enzyme subunit 1	5,2	38,3	12,0	1	35,9
SODC_BOVIN	SOD1	Superoxide dismutase [Cu-Zn] (EC 1.15.1.1)	5,9	15,5	9,3	1	43,1
SURF4_BOVIN	SURF4	Surfeit locus protein 4	7,6	30,4	12,0	2	91
SWP70_BOVIN	SWAP70	Switch-associated protein 70	5,8	68,9	3,0	1	42,4
TLN1_MOUSE	Tln1	Talin-1	5,8	269,7	6,7	7	575,8
TCPA_BOVIN	TCP1	T-complex protein 1 subunit alpha (TCP-1-alpha) (CCT-alpha)	5,7	60,2	11,0	2	122,8
TCPB_BOVIN	CCT2	T-complex protein 1 subunit beta (TCP-1-beta) (CCT-beta)	6,2	57,3	8,2	2	187,3
TCPD_HUMAN	CCT4	T-complex protein 1 subunit delta (TCP-1-delta) (CCT-delta) (Stimulator of TAR RNA-binding)	9,1	57,8	11,0	2	111,6
TCPE_MOUSE	Cct5	T-complex protein 1 subunit epsilon (TCP-1-epsilon) (CCT-epsilon)	5,7	59,6	3,1	1	61,7
TCPZ_BOVIN	CCT6A	T-complex protein 1 subunit zeta (TCP-1-zeta) (CCT-zeta) (CCT-zeta-1)	6,3	57,8	4,0	1	110,5
TXND5_MOUSE	TXNDC5	Thioredoxin domain-containing protein 5 precursor (Thioredoxin-like protein p46)	5,4	46,4	9,1	3	103,4

[a] Isoelectric point of listed proteins
[b] Molecular Weight of listed proteins
[c] Total sequence coverage corresponding to the peptide mass fingerprint
[d] Peptide count corresponds to the number of MS-fragmented peptides
[e] Combined score corresponding to the sum of all individual peptide frangmentation fingerprint scores

Table 2 - (Continued)

Accession	Gene symbol	Protein name	Theo. pI[a]	Theo. MW [kDa][b]	Seq.Cov.[c]	Peptide Count[d]	Combined Mascot-Score[e]
SYTC_BOVIN	TARS	Threonyl-tRNA synthetase, cytoplasmic (EC 6.1.1.3) (Threonine--tRNA ligase) (ThrRS)	6,3	83,4	2,9	2	39,6
ZO1_MOUSE	Tjp1	Tight junction protein ZO-1	6,2	194,6	3,0	2	54,4
TAGL2_BOVIN	TAGLN2	Transgelin-2	9,4	22,3	29,3	4	345,1
TERA_BOVIN	VCP	Transitional endoplasmic reticulum ATPase	5,1	89,3	14,0	4	247,6
TKT_BOVIN	TKT	Transketolase (EC 2.2.1.1) (TK)	8,5	67,9	16,9	5	323,9
TCTP_BOVIN	TPT1	Translationally-controlled tumor protein (TCTP)	4,7	19,6	12,8	1	70,7
TSPO_BOVIN	TSPO	Translocator protein	9,7	18,9	23,0	2	158,4
SSRA_BOVIN	SSR1	Translocon-associated protein subunit alpha	4,4	32,0	5,0	1	55,9
ECHB_BOVIN	HADHB	Trifunctional enzyme subunit beta, mitochondrial precursor (TP-beta) [Includes: 3-ketoacyl-CoA thiolase (EC 2.3.1.16)	9,9	51,3	5,5	1	33,3
TPIS_BOVIN	TPI1	Triosephosphate isomerase (EC 5.3.1.1) (TIM) (Triose-phosphate isomerase)	6,5	26,5	5,6	1	104,3
TPP1_BOVIN	TPP1	Tripeptidyl-peptidase 1 precursor (EC 3.4.14.9) (Tripeptidyl-peptidase I) (TPP-I) (Tripeptidyl aminopeptidase)	6,0	61,2	6,0	1	47,8
TPM3_RAT	Tpm3	Tropomyosin alpha-3 chain	4,8	29,0	14,0	4	157,7
TPM4_HUMAN	TPM4	Tropomyosin alpha-4 chain (Tropomyosin-4) (TM30p1)	4,5	28,4	10,1	3	126,8
SYWC_BOVIN	WARS	Tryptophanyl-tRNA synthetase, cytoplasmic	5,5	53,8	10,0	2	183,7
TBA1A_MOUSE	TUBA1A	Tubulin alpha-1A chain	4,9	50,1	35,0	10	1013
TBA1B_BOVIN	TBA1B	Tubulin alpha-1B chain	4,9	50,1	35,0	10	1025,5
TBB2C_HUMAN	TUBB2C	Tubulin beta-2C chain (Tubulin beta-2 chain)	4,6	49,8	31,7	10	713
TBB3_MOUSE	TUBB3	Tubulin beta-3 chain	4,7	50,4	17,1	7	553
TBB5_BOVIN	TUBB5	Tubulin beta-5 chain	4,8	49,6	41,0	13	1018,1
TBB6_BOVIN	TUBB6	Tubulin beta-6 chain	4,8	49,9	33,0	9	508,9
UB2L3_BOVIN	UBE2L3	Ubiquitin-conjugating enzyme E2 L3 (EC 6.3.2.19) (Ubiquitin-protein ligase L3) (Ubiquitin carrier protein L3)	9,5	17,9	14,3	1	42,1
UBA1_BOVIN	UBA1	Ubiquitin-like modifier-activating enzyme 1	5,5	117,8	23,0	11	539,3
K1529_HUMAN	KIAA1529	Uncharacterized protein KIAA1529	5,7	191,0	2,7	2	32
CN166_MOUSE	C14orf166	UPF0568 protein C14orf166 homolog	6,4	28,1	11,0	2	38
VPS29_MOUSE	VPS29	Vacuolar protein sorting-associated protein 29 (Vesicle protein sorting 29)	6,3	20,5	7,7	1	35
VPS35_BOVIN	VPS35	Vacuolar protein sorting-associated protein 35	5,3	91,7	10,0	2	37,9
VASP_BOVIN	VASP	Vasodilator-stimulated phosphoprotein (VASP)	9,5	40,3	6,5	2	49,9
ACADV_BOVIN	ACADVL	Very-long-chain specific acyl-CoA dehydrogenase, mitochondrial precursor (EC 1.3.99.-) (VLCAD)	9,4	70,6	4,3	2	81,8
VIGLN_MOUSE	HDLBP	Vigilin (High density lipoprotein-binding protein) (HDL-binding protein)	6,4	141,7	3,4	2	35,2
VIME_BOVIN	VIM	Vimentin	5,1	53,5	43,0	25	1890,4
VINC_MOUSE	Vcl	Vinculin (Metavinculin)	5,7	116,5	17,4	8	501,6
VDAC2_BOVIN	VDAC2	Voltage-dependent anion-selective channel protein 2 (Outer mitochondrial membrane protein porin 2)	8,7	31,6	6,8	1	30,6
VWF_BOVIN	VWF	von Willebrand factor (Fragment)	5,4	102,5	12,0	4	334,1
VATA_BOVIN	ATP6V1A	V-type proton ATPase catalytic subunit A	5,4	68,3	18,0	1	35,1

[a] Isoelectric point of listed proteins
[b] Molecular Weight of listed proteins
[c] Total sequence coverage corresponding to the peptide mass fingerprint
[d] Peptide count corresponds to the number of MS-fragmented peptides
[e] Combined score corresponding to the sum of all individual peptide fragmentation fingerprint scores
